# Natural Bioactive Compounds Targeting Epigenetic Pathways in Cancer: A Review on Alkaloids, Terpenoids, Quinones, and Isothiocyanates

**DOI:** 10.3390/nu13113714

**Published:** 2021-10-22

**Authors:** Nasreddine El Omari, Saad Bakrim, Mohamed Bakha, José M. Lorenzo, Maksim Rebezov, Mohammad Ali Shariati, Sara Aboulaghras, Abdelaali Balahbib, Mars Khayrullin, Abdelhakim Bouyahya

**Affiliations:** 1 Laboratory of Histology, Embryology, and Cytogenetic, Faculty of Medicine and Pharmacy, Mohammed V University, Rabat P.O. Box 1014, Morocco; nasrelomari@gmail.com; 2 Molecular Engineering, Valorization and Environment Team, Polydisciplinary Faculty of Taroudant, Ibn Zohr University, Agadir 80000, Morocco; s.bakrim@hotmail.com; 3 Biotechnology and Applied Microbiology Team, Department of Biology, Faculty of Science, Abdelmalek Essaadi University, Tetouan 93002, Morocco; bakha.mohamad@gmail.com; 4 Centro Tecnológico de la Carne de Galicia, Rúa Galicia No. 4, Parque Tecnológico de Galicia, San Cibrao das Viñas, 32900 Ourense, Spain; 5 Área de Tecnología de los Alimentos, Facultad de Ciencias de Ourense, Universidad de Vigo, 32004 Ourense, Spain; 6 V. M. Gorbatov Federal Research Center for Food Systems of Russian Academy of Sciences, 26 Talalikhina St., 109316 Moscow, Russia; rebezov@yandex.ru; 7 Department of Scientific Research, School of Agricultural Sciences, Liaocheng University, 34 Wenhua Road, Liaocheng 252000, China; shariatymohammadali@gmail.com; 8 Department of Scientific Research, K.G. Razumovsky Moscow State University of Technologies and Management (The First Cossack University), 73, Zemlyanoy Val St., 109004 Moscow, Russia; khairullin-mars@ya.ru; 9 Physiology and Physiopathology Team, Department of Biology, Mohammed V University, Rabat P.O. Box 1014, Morocco; sara.aboulghras@gmail.com; 10 Laboratory of Biodiversity, Ecology, and Genome, Faculty of Sciences, Mohammed V University, Rabat P.O. Box 1014, Morocco; balahbib.abdo@gmail.com; 11 Laboratory of Human Pathologies Biology, Department of Biology, Faculty of Sciences and Genomic Center of Human Pathologies, Mohammed V University, Rabat P.O. Box 1014, Morocco

**Keywords:** cancer, natural compounds, epigenetic, epidrugs, DNMT

## Abstract

Cancer is one of the most complex and systemic diseases affecting the health of mankind, causing major deaths with a significant increase. This pathology is caused by several risk factors, of which genetic disturbances constitute the major elements, which not only initiate tumor transformation but also epigenetic disturbances which are linked to it and which can induce transcriptional instability. Indeed, the involvement of epigenetic disturbances in cancer has been the subject of correlations today, in addition to the use of drugs that operate specifically on different epigenetic pathways. Natural molecules, especially those isolated from medicinal plants, have shown anticancer effects linked to mechanisms of action. The objective of this review is to explore the anticancer effects of alkaloids, terpenoids, quinones, and isothiocyanates.

## 1. Introduction

In recent decades, cancer has been viewed as a complex disease affecting countries around the world. In fact, the incidence of cancer is increasing despite decisive technological progress, in particular genomic, transcriptomic, proteomic, and epigenomic aspects. Cancer induction can occur through several internal and external risk factors [1,2]. These risk factors can create instability in cells, which predisposes them to transform into tumor cells. Indeed, the transformation of normal cells into tumor cells is now defined as a loss of cellular memory (loss of identity), which designates the maintenance of its state of differentiation [3].

The maintenance of cellular memory is ensured by epigenetic marks which designate the set of changes regulating gene expression without affecting the physical sequence of DNA. Epigenetic changes include DNA methylation, histone modification, chromatin remodeling, and the action of mRNA and ncRNA. All of these changes are provided by modifiers (enzymes) that catalyze these reactions in specific ways such as DNA methyltransferases (DNMT), histone deacetylase (HDAC), and HAT.

Recently, the link between the disruption of epigenetic changes, loss of cellular memory, and tumor transformation has been very well demonstrated and correlated. Moreover, given the reversibility of all epigenetic changes, it is possible to modify the target disturbances by using the inhibitors and activators of the enzymes involved in the different changes. Indeed, several molecules acting on epigenetic pathways have been introduced as anticancer drugs under the term of epidrugs. We can cite, for example, 5-azacytidine (it acts as an inhibitory molecule of DNMT), which has long been introduced as an epidrug for the treatment of certain cancers [4,5].

The search for epidrugs has been the subject of several pharmacological investigations (in vitro and in vivo) of the panoply of synthetic, hemi-synthetic, and natural molecules, by proposing them as anticancer drugs used in chemotherapy and/or in targeted therapy [6,7]. This work is a system review on the anticancer properties of four phytochemicals, alkaloids, terpenoids, quinones, and isothiocyanates, contained in plants.

**Figure 1:**
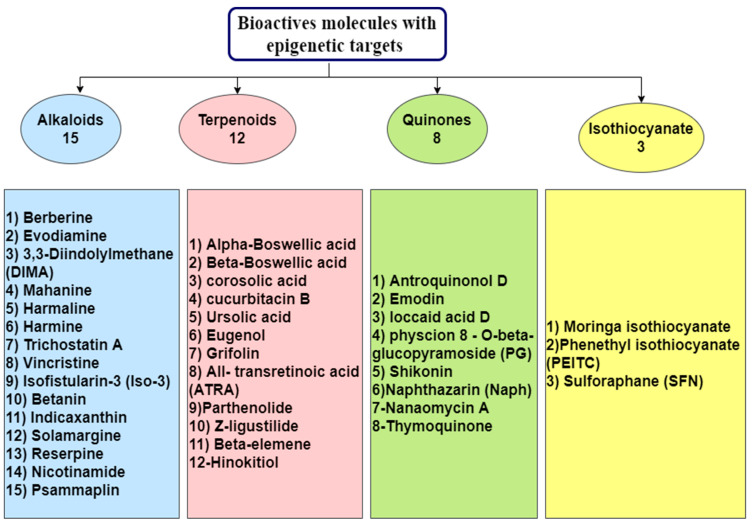
Number of molecules showed anticancer effects with epigenetic targets.

All studies about the anticancer effects (targeting epigenetic) of natural compounds belonging to these families were highlighted. Molecules (belonging to four families) showed anticancer effects with epigenetic targets are summarized in Figure 1.

## 2. Anticancer Effects of Alkaloids with Epigenetic Targets

Selected from a wide variety of natural herbs and medicinal plants, including those belonging to Leguminosae, Ranunculaceae, Loganiaceae, Papaveraceae, and Menispermaceae, alkaloids are considered one of the most important chemical compounds, representing a rich source of bioactive molecules [8]. Due to their enormous anticancer activity, Berberine, Evodiamine, 3,3′-Diindolylmethane, Harmalin, Harmine, Indicaxanthin (Ind), Isofistularin-3, Betanin, Mahanine, Nicotinamide (NA), Psammaplin, Reserpine, Solamargine, Vincristine, and Trichostatin A were chosen by many investigators to study their role in epigenetic regulation (Table 1) for the future production of drugs to prevent cancer [9].

### 2.1. Berberine

**Figure 2:**
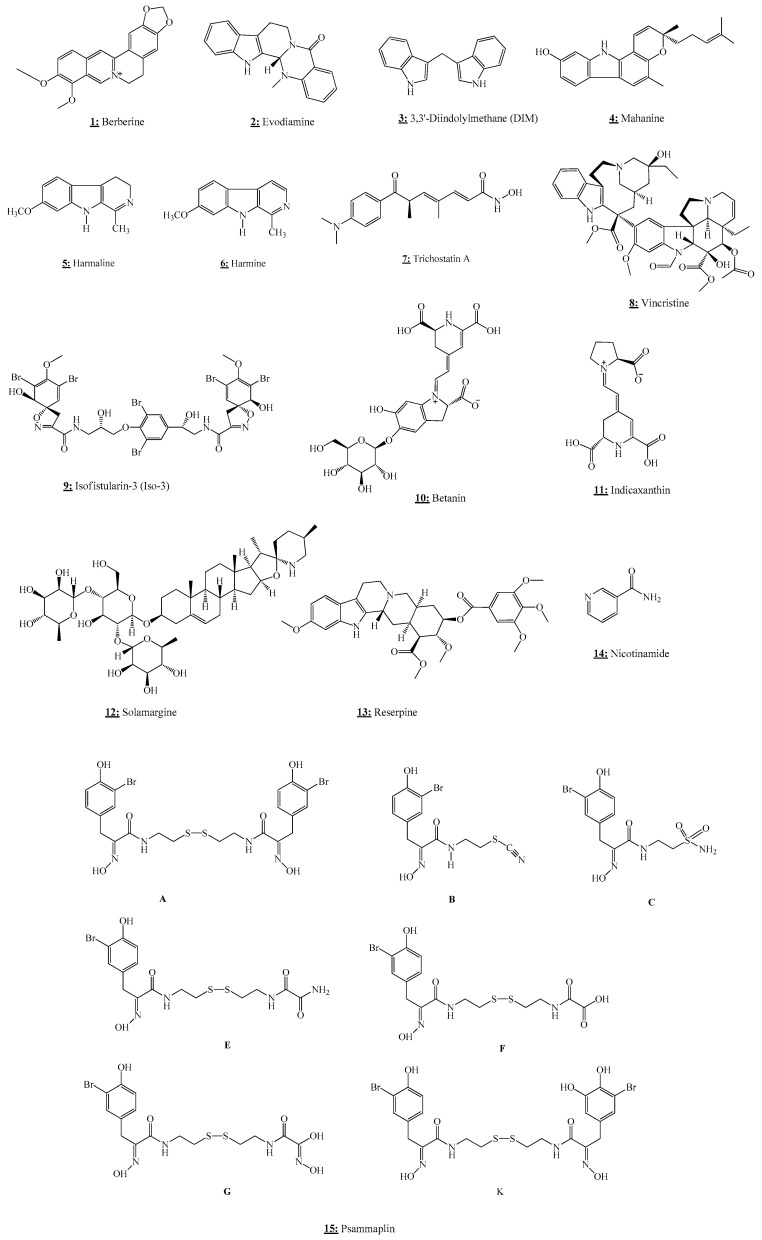
Chemical structures of alkaloids.

Berberine (BBR) is a yellow alkaloid (Figure 2) widely isolated from natural herbs and long used in Chinese herbal medicine [46]. It exhibits a wide range of pharmacological and biochemical activities, such as anti-diabetes, anti-inflammatory, antibacterial, anti-ulcer, and can also be used in the treatment of vessel expansion and in the prevention of myocardial ischemia-reperfusion injury [47].

To assess the role of berberine in epigenetic modulation, Qing et al. [10] revealed that BBR suppresses the expression of DNMT1 and DNMT3B through hypomethylation of TP53 by changing the DNA methylation level and the alteration of the p53-dependent signal pathway in U266 cells using epigenetic chromatin modification enzymes, PCR array, gene expression microarray, RT-PCR, and bisulfite sequencing. Wang et al. [11] found that Lysine-N-methyltransferase was the putative target of BBR by affecting the enzymes involved in histone acetylation and methylation using RT-PCR and western blotting analyses. The results of epigenetic chromatin modification enzymes PCR array showed up-regulation of histone acetyltransferase CREBBP and EP300, histone deacetylase SIRT3, histone demethylase KDM6A as well as histone methyltransferase SETD7, and down-regulation of histone acetyltransferase HDAC8, histone methyltransferase WHSC1I, WHSC1II, and SMYD3. In addition, treatment with BBR induced cytotoxicity and apoptosis in HL-60/ADR and KG1-α cells.

On the other hand, using an in vitro model of malignancy induced by TGF-β1, the expression levels of DNMT1, DNMT3A, DNMT3B and miR-152, miR-429, and miR-29a were notably increased after treatment with BBR and evodiamine for 24 h. These findings showed that BBR and evodiamine have beneficial effects on the interplay between DNMTs and target microRNAs in induced malignancy transformation of the colon by TGF-β1, which provides epigenetic evidence for the prevention and treatment of colorectal cancer [12].

Zheng et al. [13] demonstrated the role of BBR on the activity and expression of DNMT1 in human non-small cell lung cancer (NSCLC) cells (A549 and H1975). Consequently, BBR not only induced the inhibition of DNMT1 mRNA, protein, and promoter activity, but also reduced 3-phosphoinositide-dependent protein kinase-1 (PDPK1) and transcription factor SP1 protein expressions as well as the inhibition of growth, migration, invasion, and induction of cell cycle arrest in lung cancer cells. Furthermore, the combination of BBR with metformin enhanced the effects of BBR on the inhibition of DNMT1 gene expression through the interaction of SP1 and PDPK1.

In the context of highlighting the epigenetic potential of BBR against cerebral ischemia, Pang et al. [14] found that BBR decreases the expressions of DNMT1 and DNMT3a and reduces methylation of the PPARγ promoter region, regulates the expression of the peroxisome proliferator-activated receptor (PPARγ), and also increases PPARγ expression during cerebral ischemia-reperfusion.

The botanical alkaloid BBR has also been identified as a novel drug for the treatment of multiple myeloma (MM) through targeting the molecular mechanism UHRF1 (ubiquitin-like with PHD and RING Finger domains 1). Indeed, BBR killed MM cells in vitro and prolonged the survival of mice bearing MM xenografts in vivo. BBR can bind directly to the tandem Tudor domain and plant homeodomain (TTD-PHD domain) to induce its degradation via the ubiquitin-proteasome system, resulting in the upregulation of several tumor suppressor genes and inhibiting cell growth in both in vivo and in vitro [15].

The outcomes of these investigations have proven that BBR could play a pivotal role as an epi-drug in the interaction between epigenetics and different hallmarks of cancer.

### 2.2. Evodiamine

Evodiamine (EVO) is a major bioactive compound (Figure 2) derived from the unripe dry fruit *Evodiae fructus* (*Evodia rutaecarpa* Benth., Rutaceae) [48]. This natural alkaloid is a popular herb widely used in traditional Chinese medicine and possesses several pharmacological activities, including anti-allergenic, anti-obesity, anti-ulcerogenic, analgesic, anti-cancer, and neuroprotective effects [49].

Using a murine model of urethane-induced lung cancer and two cell models of NSCLC (A549 and H1299), Su et al. [50] demonstrated the modulatory role of EVO by using a DNMT inhibitor to investigate the role of NOTCH3 signaling in the anti-lung cancer effects of EVO. They found that EVO potently inhibits NOTCH3 signaling by activating DNMTs-induced NOTCH3 methylation. Therefore, they suggest that EVO, a novel NOTCH3 methylation stimulator, significantly suppresses lung carcinogenesis by inhibiting NOTCH3 signaling [50].

Concerning the study of the effect of EVO on epigenetic alterations, Huang et al. [12] tested the intervening effect of EVO and BBR in the interaction between DNMTs and target miRNAs using a model of malignant transformation induced by TGF-β1. They revealed that EVO and BBR had a preponderant effect on the expression of DNMTs after treatment with these two alkaloids for 24 h, in particular, increased expression of DNMT1, DNMT3A, DNMT3B and miR-152, miR-429, and miR-29a. Accordingly, they suggest that EVO and BBR could be therapeutic agents for the development of early treatment and prevention of colorectal cancer.

### 2.3. 3,3′-Diindolylmethane

3,3′-Diindolylmethane (3,3′-Methylenedi(1H-indole) (3-(1H-Indol-3-ylmethyl)-1H-indole) (DIM) is a natural bioactive alkaloid (Figure 2), derived from the digestion of indole-3-carbinol, widely found in cruciferous vegetables, including cauliflower, cabbage, broccoli, and Brussels sprouts [51].

DIM is tested in multiple clinical trials, such as breast, cervical, and prostate cancers, and has been shown to exhibit anti-cancer properties in various in vivo and in vitro models treated with carcinogens [52]. Using TRAMP-C1 cells and a TRAMP mouse model, Wu et al. [52] discovered the epigenetic modulation in vitro and in vivo of this substance. In an in vitro experiment, they found that DIM suppresses DNMT expression and reverses the CpG methylation status of Nrf2, whereas in the in vivo study, TRAMP mice treated with this molecule reduced tumorigenesis with a low incidence of metastases. Therefore, they recorded that DIM is a potent chemopreventive agent against prostate cancer and that epigenetic modifications in CpG involving Nrf2 may represent a prospective pathway by which DIM exerts its chemopreventive activities. In another study investigating the possible epigenetic mechanism of DIM [16], the effects of sulforaphane (SFN) and DIM on promoter methylation in prostate cancer cells and normal prostate epithelial cells were investigated. The authors found that these two compounds decrease the expression of the DNMT gene and cause different alterations in the DNA methylation profile depending on the prostate cell line, but they share common genetic targets within a single cell line. They also showed that DIM and SFN reverse cancer-associated DNA methylation alterations in LnCAP cells. The results of these investigations may provide new insights into the epigenetic pathways by which DIM exhibits its chemopreventive properties against cancer.

### 2.4. Harmalin

Harmalin (7-méthoxy-1-méthyl-4,9-dihydro-3H-β-carboline) (Figure 2) is a natural alkaloid isolated from the seeds of *Peganum harmala* L. (Zygophyllaceae) as well as from the hallucinogenic beverage ayahuasca [53]. It is the partially hydrogenated form of harmine and has been used traditionally as a medicine to treat certain diseases or as an odorant for vapors during certain spiritual and cultural rituals. It is used as an inverse agonist of the GABA-A receptors and induces a stimulating effect on the central nervous system, loss of coordination, agitation, and paralysis at high doses [54].

To demonstrate the possible use of harmalin as an epi-drug, a study by Nikkhoo et al. [17] was conducted to evaluate its effect on Dnmt1 gene expression and hypomethylation of the P15 promoter in the NB4 cell line. They observed that harmalin showed a dose and time-dependent antiproliferative activity on the NB4 cell line after 48 h of treatment with harmalin. They also discovered, using real-time PCR, that harmalin (15 μg/mL) induces hypomethylation of the P15 gene promoter, decreases gene expression of DNMT1, and increases P15 gene expression in the NB4 cell line. As a result, harmalin could play a central role in epigenetic mechanisms as a potential therapeutic approach, either as monotherapy or as an adjunct to drugs commonly used in the management of acute promyeloid leukemia.

### 2.5. Harmine

Harmine (7-Methoxy-1-methyl-9H-pyrido[3,4-b]indole) is a beta-carboline alkaloid found in herbal remedies such as *Peganum harmala* which has been used in folk medicine for the treatment of several diseases due to its various pharmacological properties such as antitumor, antiplasmodial, antileishmanial, antifungal, antimutagenic, antimicrobial and hallucinogenic properties, but is also reported to have a large spectrum of psychoactive activities [55,56,57].

Regarding the demonstration of the potential epigenetic effect of harmine, some investigations have been carried out [18,19].

Aghide et al. [18] investigated the role of harmine on the expression of two genes, DAPK and P16 (hypermethylated in some hematological disorders such as hematologic malignancy), in the leukemic cell line HL 60. Their finding indicates that harmine reduced cell proliferation in different concentrations and markedly up-regulates DAPK expression at 102.4 µg/mL. Nevertheless, harmine did not show any significant effect on P16.

In another study using the human promyelocytic NB4 cell line, Oodi et al. [19] elucidated the modulatory impact of harmine on the expression of DNMT1. Results showed that harmine therapy (25.6 µg/mL) resulted in the inhibition of DNMT1 mRNA expression, which led to DNA hypomethylation and reactivation (Harmine, 51.2 µg/mL). They also observed the inhibition of cell proliferation in NB4 cells at all concentrations tested in a time and dose-dependent manner. These combined results support the hypothesis that harmine may exert a potential epigenetic action against the leukemia cell line.

### 2.6. Indicaxanthin

Indicaxanthin (Ind) is a bioavailable alkaloid (Figure 2) and bioactive betalain pigment derived from Opuntia ficus-indica fruit [58]. This natural multi-target compound has been the subject of in-depth studies due to its broad spectrum of pharmacological properties such as anti-inflammatory, neuro-modulatory, anti-proliferative, and pro-apoptotic effects [59]. Using colorectal cancer cell lines (CACO2, LOVO1, DLD1, HT29, and HCT116) Naselli et al. [20] explored the influence of Ind on DNA methylation and its possible epigenetic modulation. On the one hand, they found that Ind was able to increase DNMT gene expression, inhibit DNTM activity, and enhance the expression of genes associated with DNA demethylation. On the other hand, Ind exhibited anti-proliferative activity in all cell lines, except HT29. Demethylation was induced by Ind in the promoters of certain methylation-silent onco-suppressor genes implicated in colorectal carcinogenesis (p16^INK4a^, GATA4, and ESR1), but Ind didn’t impact the methylation pattern in other basically hypermethylated genes, including SFRP1 and HPP1. Furthermore, Ind was found to bind stably to DNMT1 at the catalytic site in the molecular silico modeling process. These findings showed the possible epigenetic effect of Ind in preventing colorectal cancer which requires the regulation of DNA methylation mechanisms.

### 2.7. Isofistularin-3

Isofistularin-3 (Iso-3) is a bioactive metabolite derived from the marine sponge *Aplysina aerophoba*, belonging to the group of bromotyrosine derivatives [60]. This marine alkaloid has multiple promising pharmacological effects, including anti-tumorigenic and anti-metastatic properties [61]. Based on the remarkable anticancer activity of Iso-3, Florean et al. [21] conducted an in vitro study to investigate its potential epigenetic regulation. They showed that the Iso-3 treatment significantly decreases cell proliferation (1–50 μM; 24–72 h) and stimulates growth arrest of cancer cells in G0/G1 (5–50 μM; 24 h), with an increase in p21 and p27 expression and a reduction in cyclin E1, PCNA, and c-myc levels. Furthermore, they discovered that Iso-3 alters the aryl hydrocarbon receptor (AHR) promoter methylation, increases the AHR expression in RAJI cells (25 μM; 72 h), and suppresses the growth of a large panel of cancer cell lines, with GI_50_ values between 7.3 and 14.8 μM. They also noticed that Iso-3 reduced DNMT1 protein levels in RAJI cells but did not impact the methylation in other tested cell lines. In addition, they observed that Iso-3 causes both morphological changes and autophagy in RAJI cells, mediates caspase-dependent and independent cell death, and sensitizes to TRAIL in cancer cells. These investigations revealed that Iso-3 acts as a DNA demethylating agent with an effect on cancer epigenetics which allowed it to have a significant antiproliferative potential against cancer cell lines.

### 2.8. Betanin

Betanin (Figure 2), also called phytolaccamin or betalain (betanidin 5-O-β-D-glucoside), is a red pigment belonging to the betacyanin family. It is widely distributed in food sources such as beetroots, *Beta vulgaris* L., cactus fruits, red swiss chard, pitahaya, and amaranth. As a food additive, betanin is approved as a natural red food coloring, and its E number is E162 [62].

This natural alkaloid is used as a colorant in cosmetics and pharmaceuticals and exhibits various biological activities such as a scavenger of reactive oxygen species, antioxidant, prevents DNA damage and LDL oxidation, and has also shown potential effects in lowering blood pressure [63,64].

Paluszczak et al. [22] assessed the role of betanin on the activity and expression of DNMTs in the epithelial breast cancer MCF7 cell line, as well as its effect on DNA and histone H3 methylation. The DNMT activity was inhibited by betanin but did not influence the methylation pattern or the expression of *RASSF1A*, *GSTP1* or *HIN-1*. The global methylation of histone H3 was also unchanged. The authors of this work also observed that betanin showed no effect on DNMT1 transcription or DNMT1 protein level.

### 2.9. Mahanine

Mahanine (MH) (3,5-dimethyl-3-(4-methylpent-3-enyl)-11H-pyrano[3,2-a]carbazol-9-ol) is a carbazole phytochemical alkaloid (Figure 2) purified from *Murraya koenigii* leaves and the edible part of *Micromelum minutum*, which have been employed for a typical aroma in a variety of Indian foods and some Asian vegetables [65]. This bioactive compound is characterized by a wide variety of biological properties, including antimicrobial activity, cytotoxicity, antimutagenicity, and other physicochemical activities [66].

On the other hand, Jagadeesh et al. [23] sought to assess the possible epigenetic role of MH in human prostate cancer cells. They discovered that MH exerts a potent antiproliferative effect in prostate cancer cells by inducing the expression of an epigenetically silenced gene RASSF1A by inhibiting DNMT activity. RASSF1A also contributes to the suppression of the pivotal cell cycle modulator, cyclin D1, which subsequently represses cell proliferation and its ability to be invasive in prostate and other cancer cells.

In the same context, Agarwal et al. [24] were interested in identifying whether one or more DNMTs are implicated in the restoration of RASSF1A expression by MH. They found that MH treatment could play a key role in down-regulating DNMT1 and DNMT3B in prostate cancer cells, but not DNMT3A, via the ubiquitin-proteasome mechanism, particularly, the inactivation of Akt that mediates demethylation of the RASSF1A promoter.

These results provide evidence that the epigenetic regulation activity of MH may be considered a critical approach to prevent prostate cancer when RASSF1A expression is silenced.

### 2.10. Nicotinamide

Nicotinamide (NA), also known as niacinamide (Figure 2), is a pyridinecarboxamide and a pyridine alkaloid derived from nicotinic acid and isolated from the fungus *Lactarius subplinthogalus* [67]. It is found in foods, including fish, meat, yeast, milk, mushrooms, nuts, and green vegetables [68]. It is widely used as a dietary supplement and medication [69].

NA is a precursor of nicotinamide adenine dinucleotide phosphate (NADP) and nicotinamide adenine dinucleotide (NAD), which catalyzes enzymatic reactions. This amide derivative of vitamin B_3_ is able to exert several biological activities such as an anti-inflammatory agent, a metabolite, a cofactor, an antioxidant, a poly (ADPribose) polymerase (PARP) inhibitor, and a neuroprotective agent. It also has a role to treat some diseases, including psoriasis, schizophrenia, pellagra, and type I diabetes [70].

In the purpose to develop a new breast cancer prevention strategy, Jafary et al. [25] focused on the potential epigenetic effect of NA and valproate in the human breast cancer cell line MCF-7. They observed that combined therapy with nicotinamide and valproate suppressed the viability of MCF-7 cells, reduced cell activity, inhibited cell proliferation, as well as up-regulated p16 and p21. Additionally, since histone acetylation is a key factor in epigenetic modifications, MCF-7 cells treated with nicotinamide and valproate showed elevated levels of acetylated histone H3 using western blot analysis. It seems obvious, from the results obtained, that a combination treatment of valproate and nicotinamide has significant antitumor activity and may constitute a promising route against human breast cancer.

In an in vivo model, Tian et al. [26] were interested to evaluate the possible impact of maternal NA supplementation in inducing fetal epigenetic changes, mainly the effect on mRNA expression levels of nicotinamide N-methyltransferase (NNMT), DNMT1, a-fetoprotein (AFP), and tumor protein p53 (Tp53). The results showed that NA supplementation resulted in a reduction in placental and fetal liver genomic DNA methylation and genomic uracil contents in the fetal liver, placenta, and brain. Furthermore, NA treatment was able to induce alterations in the mRNA expression of NNMT, DNMT1, and Tp53 in the fetal liver and placenta. In this research, they also found that high-dose NA supplementation led to an increase in the level of fetal hepatic Afp mRNA (at 4 g/kg). Based on these results, it is suggested that alterations in fetal epigenetic modification and DNA base composition can be induced by NA supplementation and that maternal NA intake may also be involved in the early development of epigenetic disorders in the offspring.

From another study, Tiwari and Gupta [27] investigated in an in vivo model the epigenetic potential of natural chemopreventive/antitumor compounds such as NA, butyric acid (BA), and calcium glucarate (CAG) in combination or individually. They discovered that NA was able to prevent tumor growth, but that protection was greatly enhanced when combined with BA and CAG. Furthermore, NA therapy resulted in a significant up-regulation of HDAC, DNMT, promoter methylation of miR-203 at 4 or 16 weeks, as well as down-regulation of miR-203 levels at 16 weeks. On the other hand, NA inhibited damage to gene expression (after 16 weeks), but the co-association with BA and CAG had a greater impact than that of the compound alone. The results obtained suggested a new chemopreventive efficiency of this co-administration by regulating miR-203 activity through the modulation of epigenetics or biogenetics in a time-dependent manner in tumor growth.

### 2.11. Psammaplins

Psammaplins(*N*,*N*-(dithiodi-2,1-ethanediyl)bis[3-bromo-4-hydroxy-a-(hydroxyimino)-benzenepropanamide) are natural marine products (Figure 2) found in some marine sponges. They are bromotyrosine-derived, first isolated from *Psammaplinaplysilla* sponge, revised to sponge *Pseudoceratina purpura,* which contains oxime and disulfide moieties [71]. In 1987, psammaplin A was the first bioactive metabolite isolated. Subsequently, biprasin, psammaplin C, psammaplin E, psammaplin F, psammaplin G, and psammaplin K have also been identified [72]. Psammaplin A and its several derivatives are known to have a broad spectrum of pharmacological activities, especially in terms of antibacterial, insecticidal, and anticancer activities [71]. In addition to this, psammaplin A was reported to be a potent inhibitor of the activities of several key enzymes in eukaryotic and prokaryotic systems, including those implicated in the epigenetic control of gene expressions such as HDACs and DNMTs [30,73].

**Figure 3:**
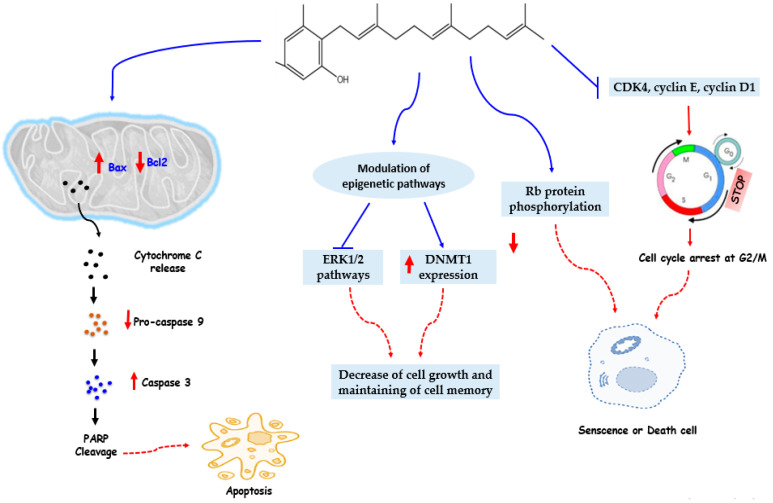
Anticancer mechanisms of psammaplins with epigenetic targets.

To understand the role of sponge-derived bromotyrosine bisulfides and their congeners as HDAC and DNMT inhibitors, Piña et al. [28] investigated several psammaplin A and its derivatives using an in vitro cell proliferation assay and an HDAC enzyme inhibition assay. The results showed that this class of anticancer products acts as dual suppressors of HDAC and DNMT. DNMT was inhibited by psammaplin A (4) and psammaplin F (11) with mild cytotoxicity, and HDAC was inhibited by psammaplin A (4) and psammaplin F (10). In the same context, Ahn et al. [29] examined the anti-proliferative effect of psammaplin A as a HDAC suppressor, measured levels of acetylated histone protein and HDAC protein, and finally assessed the pivotal role of psammaplin A on apoptosis, cell cycle arrest, and expression of tumor inhibitor genes in the human endometrial Ishikawa cancer cell line. They observed that psammaplin A derived from the two sponges, *Poecillastra wondoensis* and *Jaspis* sp., exerts a potent epigenetic regulator. It led to the inhibition of the proliferation of endometrial cancer cells treated with psammaplin A in a dose-dependent manner and induced the accumulation of acetylated histones and reduced the level of HDAC. Furthermore, it contributed to upregulation of the expression of cyclin-dependent kinase (CDK) inhibitor p21^WAF1^, as well as down-regulation of the expression of pRb, cyclins, and CDKs, which led to the induction of cell cycle arrest but also to the increase in the cellular proportion in the G1 phage and the G2/M phage detected by flow cytometry (Figure 3).

In another study, Baud et al. [30] identified the mode of action and the enzymatic specificity of psammaplin A against its epigenetic targets. Using human cancer cell lines, they demonstrated that psammaplin A (11c) had potent activity against HDAC1 in vitro (IC_50_ = 0.9 nM). Concerning its enzymatic selectivity, they found that psammaplin A exhibits high isoform selectivity, being 360-fold selective for HDAC1 over HDAC6 and more than 1000-fold less potent against HDAC7 and HDAC8. In addition, this natural compound revealed significant cytotoxicity in A549, MCF7, and W138 cells correlating with HDAC inhibition. Besides, treatment with psammaplin A resulted in the up-regulation of histone acetylation and they observed no indication that 11c acts as a DNMT inhibitor.

Based on these findings, it is clear that the use of these dual-acting marine natural products against several classes of epigenetic enzymes could be advanced as new strategies for cancer therapy.

### 2.12. Reserpine

Reserpine is a bioactive indole alkaloid (Figure 2) derived from the medicinal plant belonging to the genre *Rauvolfia,* a plant endemic to India. Reserpine is contained in the roots of six *Rauvolfia* species (*R. hookeri*, *R. micrantha*, *R. serpentina*, *R. tetraphylla*, *R. verticillata*, and *R. vomitoria*) [74]. This natural product is especially characterized by its psychopharmacological effect, particularly used to treat hypertension and psychiatric disorders [75].

Hong et al. [31] in their study hypothesized that reserpine (the main bioactive compound in *R. verticillata* extract) via an epigenetic pathway could inhibit DNMTs and control DNA methylation in a preneoplastic epidermal JB6 P+ cell line. After administration of reserpine, it was found that reserpine caused a decrease of the TPA (12-O-tetradecanoylphorbol-13-acetate)-induced JB6 cells in a dose-dependent manner. Reserpine, on the other hand, caused a demethylation effect on the first 15 CpGs of the Nrf2 promoter in JB6 P+ cells. They also discovered that reserpine inhibits the mRNA and protein expression of DNMT1, DNMT3a, and DNMT3b. Interestingly, they demonstrated that reserpine enhances cellular antioxidative activity, especially via the Nrf2 mechanism, which leads to the inhibition of TPA-induced neoplastic growth of JB6 P+ cells. These findings suggest that reserpine may play a pivotal role in the alteration of DNA demethylation and epigenetically promotes Nrf2 expression, which could be a novel pathway to prevent skin tumorigenesis.

### 2.13. Solamargine

Solamargine is a natural bioactive glycoalkaloid (Figure 2) compound found in plants of the Solanaceae family, including potatoes, eggplants, and tomatoes. It is a traditional herbal medicine isolated also from *Solanum aculeastrum* and *Solanum nigrum* L. as well as *Solanum incanum* [76]. This steroidal phytochemical alkaloid has been shown to have various pharmacological properties such as antitumor, antiviral, and anti-inflammatory effects [77].

Using human cancer lines (H1650, H1975, PC9, A549, and H1299), Chen et al. [32] aimed to underline the possible epigenetic mechanism by which the drug solamargine inhibits the development of human lung cancer cells. The results demonstrate that solamargine has a potent effect in inhibiting the expression of the DNMT1 protein and the proliferation of human lung cancer cells by decreasing the expression of the prostaglandin E_2_ (PGE_2_) receptor protein EP_4_ and activating ERK1/2 signaling. This in turn leads to a lower expression of the DNMT1 and c-Jun proteins. Solamargine, in this research, caused epigenetic changes and could be a novel strategy to inhibit lung cancer cell growth via precisely targeting EP_4_ downstream c-Jun through ERK1/2-mediated a decrease in DNMT1.

### 2.14. Vincristine

Vincristine (Figure 2), also known as leurocristine and a vinblastine analogue, is a vinca alkaloid extracted from the Madagascar periwinkle, *Catharanthus roseus* [78]. Vincristine is an anticancer drug with a wide range of biological activities used to treat various types of cancers, including acute myeloid leukemia, acute lymphocytic leukemia, neuroblastoma, Hodgkin’s disease, lung cancer, colorectal cancer, and breast cancer [79].

Furthermore, Moon et al. [33] evaluated in their research the possible epigenetic therapy of vincristine and its impact on the methylation status of the runt-related transcription factor-3 (RUNX3) gene involved in colorectal cancer, using DLD-1 colorectal adenocarcinoma cells and CCD18Co normal colon cells. They observed that treatment with vincristine was able to demethyl RUNX3 in DLD-1 cells and also caused restoration of the expression of RUNX3 mRNA in DLD-1 cells. However, DNA methylation and RUNX3 expression remained unchanged after vincristine treatment in normal CCD18Co colon cells. In order to examine RUNX3 expression levels and DNA methylation status in colorectal cancer tissues, the use of quantitative methylation-specific polymerase chain reaction (QMSP) analysis and real-time PCR identified hypermethylation of RUNX3 in 70 of 105 colorectal carcinomas (66.7%). In addition, RUNX3 mRNA expression decreased in 68 of 105 colorectal cancer tissues (64.8%).

These outcomes showed that vincristine has an epigenetic effect leading to significant demethylation of RUNX3 in colorectal adenocarcinoma cells that could constitute a novel strategy to treat colorectal cancer.

### 2.15. Trichostatin A

Trichostatin A (TSA) is a natural alkaloid (Figure 2) derivative of dienohydroxamic acid extracted from species of the bacterial genus Streptomyces [80]. To elucidate the role of histone deacetylase inhibitor TSA in T-cell leukemia Jurkat clone E6-1 cells, and its epigenetic mechanism, Januchowski et al. [34] reported using western blot analysis and quantitative real-time PCR that TSA can suppress the DNMT1 mRNA and protein expression in Jurkat T cells. They also discovered that TSA causes diminished DNMT1 mRNA stability.

**Figure 4:**
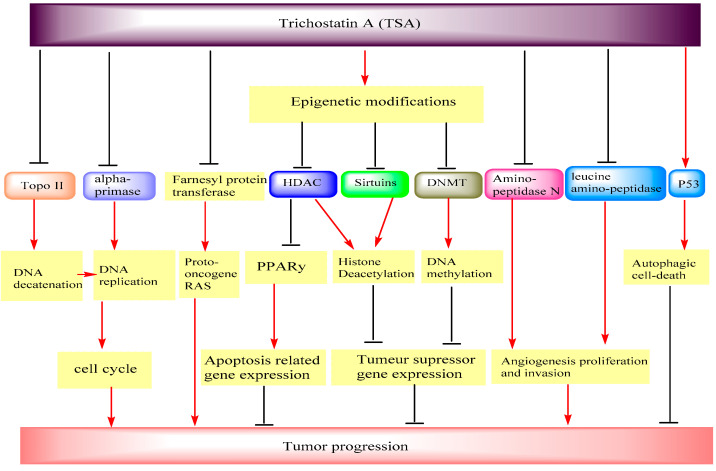
Anticancer mechanisms of trichostatin with epigenetic targets.

In a study to demonstrate the epigenetic effect of TSA, Koh et al. [35] performed research on the human melanoma cell line A2058. They observed that TSA and 5-aza-2′-deoxycytidine (Aza-dC) lead to the epigenetic modulation of sphingosine-1-phosphate (S1P) receptors in human melanoma cells and also caused a change in S1P from an inhibitor to a motility enhancer. Using quantitative PCR, treatment with TSA and Aza-dC enhanced expression of S1P_1_ and S1P_3_, associated with S1P-induced chemotaxis, and reduced expression of S1P_2_, related to motility suppression. Similarly, Vincent et al. [37], in their study, hypothesized that treatment with Aza-dC and TSA on pancreatic (PANC-1, CAPAN-1, and CAPAN-2) and gastric (KATO-III) epithelial cancer cell lines undergo epigenetic regulation. It was found that these two compounds lead to restoring the MUC4 expression in a cell-specific manner. Using chromatin immunoprecipitation and RNA interference, they demonstrated that DNMT3A, DNMT3B, HDAC1, and HDAC3 were directly implicated in MUC4 silencing by binding to its 5′-UTR in a cell-specific manner. According to their findings, TSA suppressed histone deacetylation, coupled with a high repression of MUC4 in high-expressing cells (Figure 4).

Choi et al. [38] investigated the possible epigenetic modulation mechanism of the alkaloid TSA responsible for the inhibition of *hTERT* in HCT116. Their result indicates that, for the first time, TSA was shown to have a significant epigenetic role by inducing the demethylation of site-specific CpGs on the hTERT promoter, which was due to DNMT1 down-regulation. In the second time, TSA was found to promote the CTCF binding on the hTERT promoter, resulting in hTERT suppression.

Wu et al. [40] examined the epigenetic regulation mechanism on human malignant lymphoma CA46 cells in the presence of TSA or in association with epigallocatechin-3-gallate (EGCG). After treating the cell lines, they found that TSA alone was able to inhibit CA46 cell proliferation and when it (15 ng/mL) was combined with EGCG (6 μg/mL), the proliferation of CA46 cells was diminished from 24 to 96 h. Co-treatment of TSA with EGCG was proven to down-regulate p16^INK4A^ gene methylation, which correlates with a rise in the expression of p16^INK4A^ mRNA and protein. Additionally, this combination resulted in a reactivation of p16^INK4A^ gene expression partly due to lowered promoter methylation, which may reduce CA46 cell overgrowth.

Genistein (GE), as an isoflavone product, has the ability to provide epigenetic modulations on estrogen receptor-α (ERα) reactivation-dependent breast cancer in the in vivo and in vitro models. Li et al. [41] evidenced that GE (25 μM), when combined with TSA (100 ng/mL), exhibited potential effects in preventing breast cancer; it enhanced the re-expression of ERα expression in MDA-MB-231 cells, induced the resensitization and reactivation of ERα-negative breast cancer cells to E2 and tamoxifen (TAM) antagonist, and also promoted histone remodeling changes in the ERα promoter. Further, they noted that TSA alone or in combination with GE reduced HDAC activity and had no effect on the DNMT activity. Similar to these findings, treatment with GE and TSA has been reported to inhibit cell growth at all concentrations used, down-regulate the DNMT1 gene expression after 48 and 72 h, and DNMT3a gene expression only after 72 h, but also induces apoptosis in all treatment groups tested on human hepatocellular carcinoma (HepG2) cells [81]. In the hepatocellular carcinoma Hepa 1-6 cell line, TSA could play a veritable role to prevent hepatocellular carcinoma leading to the inhibition of apoptosis and the reduction of DNMT1 expression. The relative expression of the DNMT1 gene was 0.5−0.19 [44]. In addition, on hepa-6 cells, TSA showed significant dose- and time-dependent antiproliferative effects (IC_50_~1 μM), showed significant apoptotic effects at all time periods, and significantly increased the amount of ERα gene expression [45]. The results of these studies have proven the chemopreventive properties of TSA associated with GE or TSA alone and could be considered as a potential target in the treatment of breast cancer and hepatocellular carcinoma.

On the other hand, to discover new epigenetic pathways to limit ovarian cancer malignancy, Meng et al. [42] investigated the effect of the combination of TSA and decitabine on ovarian cancer cell line SKOV3. It was found that the combined treatment with TSA and decitabine significantly limited the activity of DNMTs and HDACs, in particular the expression of HDAC1/2 and DNMT3A/3B (Figure 4), activated the acetylation of histones H3 and H4, and reduced the expression level of lysine-specific demethylase-1 (LSD1). Furthermore, the combined products inhibited the invasion and tumorigenicity of ovarian cancer cells in the in vivo models, suppressed dimethylated-H3K9, induced the transcription activity marker dimethylated-H3K4, and also suppressed migration capacity by the induction of E-cadherin and suppression of N-cadherin. Ovarian tumor progression was also repressed at least in part by the inhibition of MMP-9 and MMP-2 with the drug combination.

All obtained results of anticancer effects of natural alkaloids with epigenetic mechanisms are promising and give future perspective about their potential use in chemotherapy and chemoprevention. However, in vivo studies and clinical trials should be also investigated for confirm their activities and validate their uses.

## 3. Anticancer Effects of Terpenoids with Epigenetic Targets

**Figure 5:**
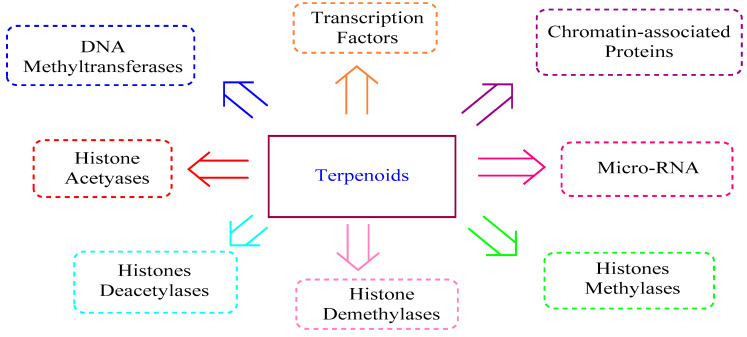
Effects of terpenoids on epigenetic modifications.

Terpenoids are the broadest range of natural products found in plants. They exhibit several biological activities and are employed in the fight against inflammation, cancer, and other infectious diseases [82]. Recently, terpenoids are being explored for use as epi-drugs with the potential to regulate epigenetic processes (Table 2 and Figure 5) [83].

### 3.1. All-Trans Retinoic Acid

**Figure 6:**
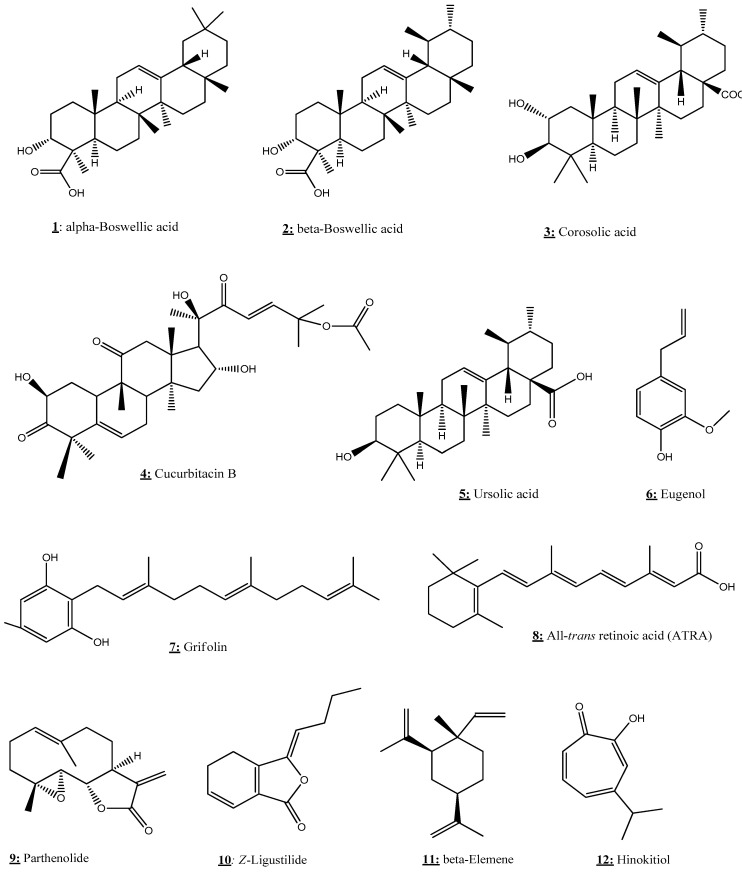
Chemical structures of terpenoids.

All-trans retinoic acid (ATRA) is a bioactive metabolite (Figure 6) of vitamin A belonging to the retinoid family. It plays a key role in a wide range of biological functions, including cell growth, organogenesis, differentiation, and apoptosis. It is considered to be an effective cancer treatment and a good chemopreventive agent. In this sense, several investigations have been conducted to highlight the epigenetic mechanisms of ATRA [84,85,86,87,88,89,90,91,92].

Using human breast cancer cell lines (MCF-7, MDA-MB-231, SK-BR3, MDA-MB-453, and HS578T), Mongan et al. [84] investigated the modulatory impact of the combination of ATRA, valproic acid, and 5-aza-2′-deoxycytidine (Aza-dC) on DNA methylation to enhance the reactivation of silenced RARβ2 in breast cancer cells. Indeed, this combination allowed to restore the expression of RARb2 in MCF-7 breast cancer cells as well as to inhibit the breast cancer cell proliferation in both ERα-positive and ERα-negative.

### 3.2. ERα-Negative

Stefanska et al. [85] evaluated the possible epigenetic effects of ATRA, vitamin D_3_, and resveratrol alone and in combination with 2-chloro-2′-deoxyadenosine (2CdA) and 9-beta-d-arabinosyl-2-fluoroadenine (F-ara-A) on the methylation and expression of retinoic acid receptor-*β*2 (RAR-*β*2) in MCF-7 and MDA-MB-231 breast cancer cell lines. They demonstrated that ATRA can methylate, at least in part, the RAR-*β*2 promoter in the tested fragment in MCF-7 cells and also induces RAR-*β*2 expression, in MDA-MB-231 cells, without any considerable effects in a combined treatment constituted only by 2CdA, ATRA, and F-ara-A. ATRA was able to improve the action of 2CdA and F-ara-A on the expression and/or methylation of RAR-*β*2. Thereby, ATRA inhibited the promoter methylation and increased the expression of RAR-*β*2 in MCF-7 cells. ATRA could also possess a beneficial effect in the reduction of phosphatase and tensin homologue (PTEN) promoter methylation in MCF-7 and MDA-MB-231 breast cancer cells [88].

Another study by Lim et al. [86] has shown that treatment with ATRA resulted in hypomethylation of the p16 and p21 promoters through the inhibition of DNMT1, 3a, and 3b. Consequently, the transcriptional activators Ets1/2 and p53 were recruited more potently to the p16 and p21 promoters, respectively. The increased regulation of these two promoters mediated by ATRA caused the induction of cellular senescence in HepG2 cells. The potential of ATRA mediated by the increase of RAR-*β*2 expression via promoter hypomethylation was implicated in the induction of cell senescence.

Another possible epigenetic effect of ATRA was tested using the androgen receptor (AR) in human prostate cancer. Liu et al. [87] showed that ATRA inhibits cell proliferation and increases HOXB13 expression in AR^−^ prostate cancer cells, leading to a reduction in the methylation level of the HOXB13 promoter. Furthermore, ATRA could alter the expression of the enhancer of zeste homolog 2 (EZH2) and DNMT3b and weaken their interactions with a HOXB13 promoter.

On the other hand, Heo et al. [89] demonstrated that this molecule can increase p14 levels via promoter hypomethylation and thus activate p53 through the p14-MDM2-p53 route in HepG2 cells. For this effect, ATRA activated p53, which modulates both the levels and activities of several apoptosis-related molecules, leading to apoptosis.

Using NSCLC cell lines (A549, NCI-H460, and HCC827), the study by Greve et al. [90] showed that a combination with ATRA and panobinostat had additive and synergistic effects, respectively, on growth inhibition and differentiation, with almost no cytotoxicity. Therefore, this combination affected histone acetylation. The combined treatment caused a decrease in the expression of phospho-ERK and phospho-AKT, while the p53 and p21^CIP1/WAF1^ proteins were both induced.

Regarding the evaluation of the epigenetic action of ATRA with another agent such as clofarabine, the results showed the inhibition of cell growth and the induction of caspase-3-dependent apoptosis in human erythroleukemic cell lines K562. Subsequently, the combinatorial products down-regulated the DNMT1 and up-regulated the CDKN1A, with a concomitant enhanced decrease in the DNMT1 protein level. As a result, ATRA and clofarabine induced a simultaneous methylation-mediated reactivation of RARB and PTEN [92].

Gao et al. [93] analyzed the role of ATRA and decitabine against elderly acute myeloid leukemia (AML). Treatment with ATRA and decitabine has been shown to exert an antitumor action which induces growth inhibition, cell cycle arrest, and apoptosis in AML cells. Additionally, they observed that ATRA and decitabine inhibited DNMT1, activated miR-34a via promoter hypomethylation, down-regulated its MYCN target, and therefore exhibited a synergistic anti-cancer action. Thus, the results indicated that ATRA could regulate epigenetic mechanisms in malignant cells and can be used as an epi-drug to prevent several types of cancer.

### 3.3. Boswellic Acid

Boswellic acid (BA) is a natural herbal (Figure 6) compound isolated from the plant *Boswellia serrata*. This bioactive triterpene, mainly used in traditional Indian medicine, has potent anti-cancer and anti-inflammatory properties. In the aim to discover the epigenetic mechanism of this compound, Shen et al. [94] performed a study using human colorectal cancer (CRC) cell lines (RKO, SW48, and SW480). The results indicated that BA treatment prevents cell growth and viability and induces the apoptosis of CRC cells. Moreover, BA induced the demethylation of several CpG loci and modulated the DNMT activity in CRC cells. These results suggest that BA could play a chemopreventive role in the treatment of CRC.

### 3.4. Corosolic Acid

Corosolic acid (CA), also called 2α-hydroxyursolic acid (Figure 6), is a natural triterpenoid found primarily in cranberries, apple peels, and blueberries. It is known for its beneficial effect to regulate the development of carcinogenesis in several types of cancers such as prostate cancer, cervical cancer, colorectal cancer, and glioblastoma [96].

Yang et al. [95] aimed to investigate the role of CA in the demethylation and reactivation of Nrf2 and CpG expression through epigenetic regulation in TRAMP-C1 prostate cells. CA treatment significantly decreased cell viability in a time- and dose-dependent manner and increased the mRNA and protein expression levels of Nrf2 and its downstream genes. Furthermore, CA decreased CpG methylation in the promoter region of Nrf2. Using chromatin immunoprecipitation (ChIP) assay, CA increased the acetylation of histone H3 lysine 27 (H3K27ac) and decreased the trimethylation of histone H3 lysine 27 (H3K27Me3) in the promoter region of Nrf2. Thereby, corosolic acid could down-regulate the expressions and activities of epigenetic modifying enzymes in TRAMP-C1 prostate cells.

Another study examined the possible epigenetic action of CA by evaluating its epigenetic effects on mouse epidermal JB6 P+ cells [96]. They discovered that treatment with this acid down-regulated the small proline-rich protein (Sprr2h) and also reversed the differentially methylated regions (DMRs) in genes like *Dusp22* (Dual specificity protein phosphatase 22) and Rassf (Ras association domain family) in JB6 P+ cells. In addition, CA modulated the CDK1 (Cyclin-dependent kinases 1) and *RASSF2* (Ras association domain family member 2) genes. These combined results suggest that CA showed marked anticancer potency in TRAMP-C1 prostate cells and mouse epidermal JB6 P+ cells, which can be due to epigenetics, including its capacity to epigenetically remediate the expression of Nrf2 and modulate global CpG methylation.

### 3.5. Cucurbitacin B

Cucurbitacin B (CuB) is a bioactive triterpene (Figure 6) compound isolated from *Trichosanthes cucumerina* L. It has been largely used in folk medicine, in particular in India, and is also characterized by a broad spectrum of pharmacological activities such as anti-inflammatory and antineoplastics [118].

Shukla et al. [97] suggested in their research that CuB might suppress DNMTs and HDAC in lung cancer due to its inhibitory effects on lung cancer (NSCLC H1299 cell lines). They produced lung cancer in human NSCLC cells and treated them with this molecule. In NSCLC H1299 cells, CuB was found to inhibit both DNMTs and HDACs at IC_50_ = 60 nmol/L. It also altered the expression of epigenetic enzymes and tumor-related genes in NNK-induced lung cancer in A/J mice. In addition, this molecule altered histone modifications in the expression of key tumor-related genes, including the p16^INK4A^, p21^CIP1/WAF1^, and hTERT promoters, which promote the inhibition of cell proliferation and apoptosis. Dittharot et al. [98] evaluated the role of CuB using breast cancer cells (MCF-7, MDA-MB-231) and breast epithelial cells (MCF-10A). They discovered that CuB increased DNMT1 and heavy methylation evident in the promotors of cyclin D1, survivin, and c-Myc, thus decreasing the expression of all of these key oncogenes. Treatment with CuB was able to inhibit cell proliferation in breast cancer cells using MTT assay and colony formation assay. These combined results suggest that CuB can be used against NSCLC and lung cancer in humans due to its potential epigenetic action.

### 3.6. Grifolin

Grifolin (Figure 6) is a natural secondary metabolite extracted from the mushroom *Albatrellus confluens*, shown to exert anti-tumor properties [119].

They noticed that the action of grifolin on human nasopharyngeal carcinoma (NPC) CNE1, CNE1-LMP1, and C666-1 cells occur notably via the attenuation of glycolytic flux and the recovery of mitochondrial OXPHOS function by inhibiting DNMT1 expression and activity as well as its mitochondrial retention in NPC cells [100]. A study by Luo et al. [99] provided clear evidence that grifolin treatment inhibits the kinase activities of ERK1/2 and avoids adhesion, migration, and invasion of high-metastatic cancer cells. Grifolin was able to downregulate the level of DNMT1 mRNA and inhibit the transcription activity of Elk1 and decrease the phosphorylation of Elk1 at Ser383 and the protein as well as its binding to the DNMT1 promoter region. The epigenetic modulation of DNMT1 function via grifolin led to inactivation of tumor metastases in a metastatic mouse model. Thus, the results showed that grifolin provides significant epigenetic mechanisms and could be considered a novel anti-tumor drug to avoid malignancy and metastasis.

### 3.7. Hinokitiol

Hinokitiol (Figure 6) is a natural bioactive terpenoid found in various medicinal and aromatic herbs. It exhibits a wide range of pharmacological activities, including anti-cancer and anti-inflammatory effects [120]. Seo et al. [101] were interested in the possible epigenetic modulation of hinokitiol, in particular on the activity and expression of DNMTs in human colon cancer cell lines (HCT-116 and SW480). Their discovery indicates, for the first time, that hinokitiol can exert DNA demethylation by reducing DNMT1 and ubiquitin-like with plant homeodomain and RING finger domain 1 (UHRF1) expression in HCT-116 cells. In addition, the results of this study showed that hinokitiol restored the mRNA expression of O6-methylguanine DNA methyltransferase (MGMT), carbohydrate sulfotransferase 10 (CHST10), and B-cell translocation gene 4 (BTG4), along with a reduction of methylation status in HCT-116 cells. Besides, hinokitiol has the capacity to increase TET1 expression and 5-hydroxymethylcytosine (5hmC) levels in both colon cancer cells.

### 3.8. Eugenol

Eugenol (4-hydroxy-3-methoxy-allylbenzene) is a natural phenolic component widely used in cosmetics and food as a flavoring product. It is found in the essential oil of various plants such as the *Lauraceae*, *Lamiaceae*, *Myristicaceae*, and *Myrtaceae* families [121]. Eugenol could exhibit chemopreventive properties against cancer and exert epigenetic modulations. Pal et al. [102] demonstrated that this natural substance, in combination with EGCG and amrogentin, could strongly induce apoptosis and inhibit cell development and colony formation, but may also down-regulate the cyclin D1 and up-regulate cell cycle inhibitors LIMD1, RBSP3, and p16 at the G1/S phase of the cell cycle. In regards to its potential epigenetic activity, eugenol induced promoter hypomethylation of LIMD1 and P16 genes following the reduced expression of DNMT1 in the human cervical cancer cell line Hela. The results of this work suggest that eugenol could play a central role in the prevention of cervical cancer.

### 3.9. Parthenolide

Parthenolide (PTL) is a sesquiterpene lactone and a secondary metabolite derived from the feverfew plant (*Tanacetum parthenium* L.). It has been shown to exhibit anti-inflammatory and anti-cancer properties, making it a prime candidate for further study and drug discovery [122].

Liu et al. [104] conducted a study to understand the role of PTL in the modulation of DNA methylation in leukemia cell lines. They found that treatment with PTL induced global DNA hypomethylation and enhanced histone acetylation in a dose- and time-dependent manner in leukemia cell lines MV4-11. PTL could also suppress *M. SssI* activity with an EC_50_ of 5.0 µM and deplete DNMT1 protein at 10 µM. In addition, PTL has been shown to down-regulate the DNMT1 transcription in a time- and dose-dependent fashion, to increase the histone acetylation without altering enzyme level as well as to up-regulate p21.

Gopal et al. [103] observed that PTL treatment exhausted HDAC1 protein without impacting other class I/II HDACs and also enhanced HDAC1 depletion and cell death via the DNA-damage-transducer ataxia telangiectasia mutated.

Liu et al. [105] in their research found that PTL inhibited DNMT1 (IC_50_ = 3.5 μM), due to possible alkylation of the proximal thiolate of Cys^1226^ of the catalytic domain by its γ-methylene lactone. Subsequently, PTL could decrease the DNMT1 expression possibly related to its SubG_1_ cell-cycle arrest or the interruption of transcriptional factor Sp1 binding to the DNMT promoter. In addition to this, PTL restored the tumor suppressor *HIN-1* gene in vitro probably due to its promoter hypomethylation.

Dai et al. [106] were interested in studying the epigenetic effect of PTL on HDAC inhibitor (HDACi) lethality in human AML cells. After treating human AML cell lines, they found that PTL prevented HDACi-induced activation of the canonical NF-κB pathway. In addition, PTL potentiated the HDACi-induced apoptosis in various human AML cell lines and enhanced the HDACi lethality in primary AML blasts. In addition, the results revealed that the activation of SAPK/JNK was shown to play a significant effect in the potentiation of HDACi lethality via PTL in human AML cells.

Ghantous et al. [107] studied the epigenetic effect of PTL on murine and human epidermal cell lines and JB6 cells. They found that PTL inhibited the growth of tumor epidermal cells in human and murine in vitro models and suppressed tumor promotion in 2D and 3D cultures. Furthermore, PTL was proven to inhibit the tumor promoter-induced NF-κB activity and modulate p21 and cyclin D1 NF-κB target genes, and also inhibit the TPA-induced tumor growth in vivo. However, PTL was a potent drug to induce the S-phase arrest in nonpromoted cells and block tumor-promoted cells at S to G2/M phases as well as to modulate the p65 binding and chromatin structure on p21 and cyclin D1 promoters regulating their gene expression.

In another study performed by Carlisi et al. [108], PTL, without combination with another product, stimulated the survival pathway Akt/mTOR and the consequent nuclear translocation of Nrf2, whereas, combined treatment with PTL and hydroxamic acid (SAHA) induced GSH depletion, a fall in ΔΨm, cytochrome c release, caspase 3 activation, and apoptosis. In addition, the results demonstrated that PTL and SAHA allowed the preservation of both hyperacetylations of histones H3 and H4 induced by HDACi and the decrease of DNMT1 expression induced by PTL. These results highlighted the epigenetic action of PTL which could be a promising drug as a chemopreventive herbal medicine to protect against tumor cells.

### 3.10. Ursolic Acid

Ursolic acid (3β-hydroxy-urs-12-ene-28-oic acid, UA) is a natural terpenoid drug with a broad spectrum of pharmaceutical activities. Ursolic acid is a secondary herb metabolite, frequently found in leaves, stem bark, or fruit peel [123]. It is largely used in traditional medicine and researchers have recently returned to this compound to study its interest as an epi-drug. In this context, [109,110,111,112,113] understood the epigenetic mechanisms of UA and its potent anticancer. After treating human hepatocellular carcinoma cells HepG2 with UA, Yie et al. [109] observed a significant inhibition of HCC cell growth and induction of apoptosis depending on the dose and time, as well as the activation of phosphorylation of AMPKα and the suppression of the protein expression of DNMT1 in a dose-dependent manner. Additionally, UA repressed the expression of transcription factor Sp1 protein. Similarly, Wu et al. [110] showed that UA limited the growth and apoptosis of human NSCLC via the SAPK/JNK-mediated reduction of DNMT1 and EZH2. The study by Kim et al. [111] found that UA inhibits the growth and the TPA-induced transformation of epidermal JB6 P+ cells. They also observed that UA upregulates Nrf2 and its downstream detoxifying/antioxidant target genes and decreases the Nrf2 promoter methylation. Moreover, UA decreased the expression of epigenetic modifying enzymes, in particular DNMT1 and DNMT3a, and HDAC1, 2, 3, and 8 (Class I) and HDAC6 and 7 (Class II), and HDAC activity. UA exhibited anti-oxidant and anti-inflammatory effects by suppressing lipopolysaccharide (LPS)-induced iNos expression. Furthermore, UA attenuated the induction of epigenetic markers (DNMT1, DNMT3a, HDAC1, and HDAC3) in leukocytes, mediated by LPS, and also increased the expression levels of Ho1, Nqo1, and Ugt1a1 [112].

In the in vitro model targeting USP7, UA showed potent inhibitory activity against USP7 at IC_50_ = 7.0 ± 1.5 μmol/L. UA might interact with USP7 in RPMI8226 human myeloma cells and could inhibit myeloma cell proliferation (IC_50_ = 6.56 μmol/L), accompanied by reductions in USP7 substrates such as MDM2, UHRF1, and DNMT1 related to the inhibition of USP7. In addition, UA occupied the ubiquitin-binding pocket of USP7, with the 17-carboxyl group and 3-hydroxyl group playing a vital role in the USP7-UA interaction [113]. These findings suggest a potential epigenetic action of the terpenoid UA against cancer.

### 3.11. Z-ligustilide

Z-Ligustilide (3-butylidene-4,5-dihydrophthalide, LIG) is a principal active component of several medicinal plants of the Umbelliferae family [124]. It is one of the bioactive products of *Radix Angelicae* *Sinensis* (RAS), largely used for centuries as a dietary supplement in traditional Chinese medicine. Using prostate tumors in TRAMP C1 cells, Su et al. [114] investigated the epigenetic modulation of LIG on DNA methylation in Nrf2 gene expression. They found that LIG and RAS markedly reduced the relative quantity of methylated DNA in the Nrf2 gene promoter region and also suppressed DNMT activity in vitro. Moreover, LIG and RAS induced the mRNA and protein expression of endogenous Nrf2 and Nrf2 downstream target genes, such as HO-1, NQO1, and UGT1A1, and also led to a decrease in the level of methylation of the first five CpGs of the Nrf2 promoter.

On the other hand, Ma et al. [115] determined the role of LIG in inducing epigenetic changes in Erα-negative breast cancer. It was observed that LIG treatment restored the growth inhibition of TAM on ERα-breast cancer cells, reactivated ERα expression and transcriptional activity, increased the Ace-H3 (lys9/14) enrichment in the ERα promoter, reduced the enrichment of metastasis-associated protein 1 (MTA1) as well as IFN-γ-inducible protein 16 (IFI16) and HDACs on the ERα promoter, and also enhanced the Ace-H3 (lys9/14) enrichment in the ERα promoter. In addition to this, LIG was able to down-regulate MTA1, IFI16, and HDACs, resulting in the destabilization of the corepressor complex. Moreover, they reported that the combination of LIG and TAM induced apoptosis and S and G2/M phase cell cycle arrest. The results of these studies suggest that LIG may serve as a novel therapeutic agent by restoring Nrf2 gene expression through an epigenetic change in TRAMP C1 cells and may also pave new opportunities for the management of severe tamoxifen-resistant breast cancer.

### 3.12. β-Elemene

β-elemene is a natural bioactive compound extracted from the rhizome of *Curcuma wenyujin*, widely known for its several anti-tumor properties [125]. The possible epigentic action of this product has aroused the interest of many researchers [116] and [117]. Using human NSCLC cells, Zhao et al. [116] showed that β-elemene inhibits NSCLC cell growth and increases the phosphorylation of ERK1/2, Akt, and AMPKα. β-elemene has the ability to inhibit the expression of DNMT1. In addition, the potent anti-tumor activity of this natural compound could be due to the suppression of the Sp1 protein expression, which was eliminated by either ERK1/2 or an AMPK inhibitor. Wu et al. [117] demonstrated by using NPC cells that β-elemene decreased the phosphorylation of signal transducer and activator of transcription 3 (Stat3) and protein expressions of DNMT1 and EZH2. They also reported that β-elemene suppressed tumor growth by inactivating Stat3 and reduced DNMT1 and EZH2 expressions in a mouse xenograft model.

Terpenoid compounds are major compounds of essential oils and therefore the use of medicinal plants in phytotherapy could have a chemoprevention effect. However, the development of these terpenoids as epidrugs needs further investigations.

## 4. Anticancer Effects of Isothiocyanates with Epigenetic Targets

The effects of isothiocyanates on epigenetic targets in different human cancers are summarized in Table 3.

### 4.1. Moringa Isothiocyanate

**Figure 7:**
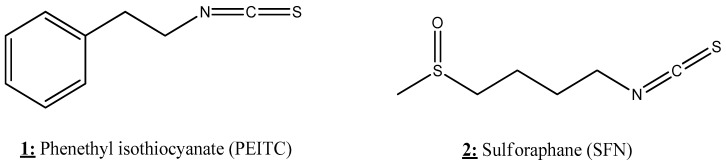
Chemical structures of isothiocyanate with anticancer effects via epigenetic targets.

Isothiocyanate (Figure 7) is a naturally occurring compound of the isothiocyanate group from Moringa oleifera, a plant known for its various biological proprieties, notably anti-inflammatory and antioxidant effects. This compound is known to induce cytotoxicity and epigenetic modifications in mouse epidermal cells. Due to its potential to up- or down-regulate gene expression, isothiocyanate has been shown to be potent in preventing the alteration of gene expression caused by TPA (12-O-tetradecanoyl-phorbol-13-acetate) inducing carcinogenesis in mouse epidermal JB6 P+ cells. In fact, TPA enhanced the inflammatory response by activation of NF-κB, IL-1, and LPS/IL-1-mediated inhibition of the retinoid X receptor (RXR) function, and generated oxidative stress by inhibiting Nrf2 (regulator of antioxidant proteins expression), as well as downregulating the tumor suppressor genes p53 and PTEN. However, isothiocyanate could repair the gene expression damage caused by TPA through the inhibition of NF-κB, IL-1, and LPS/IL-1-mediated inhibition of RXR function and by re-activation of Nrf2, p53, and PTEN [128].

### 4.2. Phenethyl Isothiocyanate

Phenethyl isothiocyanate (PEITC) (Figure 7) has been shown to be able to regulate epigenetic marks related to the progression of prostate and colorectal cancer cells. Long-term exposure to low doses (2.5 μM) of PEITC could regulate the profile of many epigenetic writers/erasers, notably PRC, histone methyltransferase (HMT), histone acetyltransferase (HAT), HDAC, and LSD as well as PcG complexes in colorectal cancer cells. These epigenetic modifications upon PEITC treatment were related to DNA hypomethylation and up-regulation of the tumor suppressor gene BMI-1, resulting in the decreased viability of cancer cells, in vitro and in vivo [127]. In addition, the combinatorial treatment of PEITC with laccaic acid (LA) showed synergistic antitumor activity on colorectal cancer cells with noticeable ability to down-regulate the expression of DNMT1 and HDAC1 [129]. In the case of prostate cancer, PEITC induces epigenetic modifications in vitro and in vivo by influencing the expression of genes related to inflammation-related TNFR signaling and PTEN/PI3K/AKT signaling [128]. Another important study showed that PEITC can reduce the invasiveness of prostate cancer. In fact, PEITC treatment leads to an increase in the expression of miR-194 that down-regulates BMP1 expression and subsequently reduces the expression of oncogenic MMP2 and MMP9 involved in tumor metastasis [112].

### 4.3. Sulforaphane

Sulforaphane (SFN) is an isothiocyanate (Figure 7) compound found in cruciferous vegetables such as broccoli (Figure 5). SFN is known for its health benefits due to its various and potent biological activities such as antioxidant and anticancer properties [151]. Numerous investigations reported that SFN is an efficient epigenetic modulator that has multiple effects on DNA methylation profiles in multiple cancer cells. This compound could reverse both hypo- and hypermethylation and subsequently increase and decrease the expression of numerous genes related to transcription, apoptosis, cell migration, and immune response. The ability of SFN to modify the epigenetic marks varies in a dose- and time dependent manner depends on cancer type, cell type, and the combinatorial agents [131,143,149].

SFN alone or in combination with other compounds (quercetin, catechin gallate, EGCG, epicatechin gallate, and green tea catechins) has been shown to induce a significant decrease in the growth and invasion of pancreatic cancer cells. In fact, SFN in combination with green tea catechins (GTC) strongly reduced ALDH1 activity in MIA- PaCa2 and BxPc-3 cells [133]. ALDH1 is an enzyme that plays a key role in the development of cancer stem cells (CSCs) [152]. SFN alone or in combination with quercetin or GTC decreased the expression of matrix metalloproteinase-2 (MMP-2) and MMP-9, which are known to be potent triggers of prostate cancer metastasis [133,153]. Moreover, SFN alone or in combination with quercetin or GTC significantly enhanced the expression of miR-let-7a, which resulted in the subsequent inhibition of K-ras expression and CSC features in pancreatic cancer cells [133]. MiR-let-7a has been reported to be potent in decreasing the proliferation and invasion of many cancer cell lines [154].

Furthermore, SFN showed a protective effect against ethanol-induced apoptosis in neural crest cells (NCCs). This activity is primarily attributed to the SFN ability to restore the epithelial-mesenchymal transition (EMT) by downregulating E-cadherin, an EMT-suppressing factor, and by up-regulating vimentin, an EMT-promoting factor. In fact, SFN could reduce the expression of E-cadherin by upregulating the expression of Snail1, known as the transcriptional repressor of E-cadherin, by decreasing H3K4me3 at the promoter regions of Snail1 [147].

SFN also possesses effective antitumor activity against the hepatocarcinoma HepG2 cell line. At the epigenetic scale, SFN up-regulates the expression of the DUSP4 tumor suppressor gene and CDK proteins and down-regulates genes related to the MAPK, WNT, and interleukin signaling pathways. These modifications were correlated with the down-regulation of HDAC5 and HDAC11 genes upon SFN [148,150]. Santos et al. [148] also showed that SFN could induce a similar impact on human primary gastric cells as that observed on hepatocarcinoma HepG2 cell. On the other hand, SFN showed significant anti-tumor activity against two NPC cell lines C666-1 and HONE-1, with in vivo inhibition of C666-1 cells. This effect was mediated by the up-regulation of WIF1 (a tumor suppressor) and the decline of DNMT1 [145].

SFN and combinatorial treatment with 5-aza-2′-deoxycytidine (DAC) showed the significant inhibition of melanoma cell growth with a marked increase in CCL5, DUSP15, and IL33 expression [146]. CCL5 is a cytokine known as a chemoattractant for natural killer (NK) cells involved in inhibiting melanoma growth [155]. In an in vivo study, the SFN anti-cancer activity against skin cancer cells in mice was mainly related to the ability of the molecule to inhibit TPA-induced neoplastic transformation via the reactivation of Nrf2 by reducing the protein expression of DNMTs and HDACs [134]. In prostate cancer cells, the decrease in DNMT expression by SFN induces re-expression of several hypermethylated genes, such as TGFBR1 and CYR61 [16], while reactivation of Nrf2 by SFN leads to Nrf2-induced NQO-1 expression, a protein that plays a key role in antioxidant protection [126,127,128,129,130,131,132,133,134,135,136,137,138,139,140,141,142,143,144,145,146,147,148,149,150,151,152,153,154,155,156].

SFN showed inhibitory effects in two colorectal cancer cell lines, RKO and HCT 116, by decreasing their growth and inducing apoptosis via epigenetic modifications, mainly by decreasing the expression and activity of HDAC. SFN has also been shown to inhibit the expression of hTERT as well as the oncogene miRNA-21 [143]. In fact, it is proven that miRNA-21, overexpressed in colorectal cancer cells, increases the expression of hTERT via the PTEN/ERK1/2 signaling pathway [157]. In addition, the chemopreventive effect of SFN in colon cancer could be due to the SFN ability to reactivate the Nrf2 expression by promoting the demethylation of the Nrf2 promoter by attenuating DNMT1 expression [149]. In lung cancer cells, SFN notably decreased the activity of many epigenetic marks, notably DNMT3a, HDAC1, HDAC3, HDAC6, and CDH1 [136,140]. It has also shown potent effects in restoring miR-9-3, hypermethylated in lung cancer A549 cells, by up-regulating H3K4me1 in the miR-9-3 promoter region [140].

Moreover, SFN alone or in combination showed excellent results against breast cancer development via its ability to induce many epigenetic modulations. SFN reduced the expression of DNMT1 and DNMT3B in three breast cancer cell lines (MCF-7, MDA-MB- 231, and SK-BR-3) with a significant increase in the expression of p21 and p27, and down-regulation of miR-23b, miR-92b, miR-381, and miR-382 [137]. SFN treatment also led to a decrease in the methylation of the PTEN and RARbeta2 promoter region and subsequently up-regulated their gene expression levels. According to Lubecka et al. [91], the combinatorial treatment of SFN with clofarabine could induce a significant decline of cancer cell growth and up-regulation of CDKN2A (a tumor suppressor gene highly hypermethylated in breast cancer cells). SFN also significantly inhibits the expression and activity of hTERT in breast cancer MCF-7 and MDA-MB-231 cells via several epigenetic modifications [130,144]. In fact, the alteration of hTERT by SFN was related to SFN-induced chromatin modification of the hTERT promoter region, notably the up-regulation of acetyl-H3, acetyl-H3K9, and acetyl-H4, and the down-regulation of trimethyl-H3K9 and trimethyl-H3K27 with considerable inhibition of DNMT1 and DNMT3a. The inhibition of hTERT was also linked to the impact of SFN on the expression of MAD1 (a repressor of hTERT) and c-MYC (an activator of hTERT) in MCF-7 and MDA-MB-231 cancer cells [130]. Additionally, SFN alone or in combination with genistein down-regulates the expression of hTERT through the decline of KLF4 [144]. In fact, KLF4 is a potent activator of hTERT, which is over-expressed in most breast cancer cells [158]. On the other hand, the combination of SFN with withaferin A induces a significant decline of DNMTs and HMT and an increase in HAT, which leads to a significant down-regulation of BCL-2, cyclin D1, CDK4, and pRB genes (involved in cell cycle), and an up-regulation of E2F, p21 (tumor suppressor), and BAX (pro-apoptotic) [138,159]. Additionally, SFN has been found able to reverse estrogen-induced metabolic changes in breast cancer MCF-7 cells [139].

In an in vivo study, a prenatal/maternal broccoli sprouts (BSp) diet rich in SFN showed remarkable preventive effects against breast cancer development in two transgenic mouse models. However, the postnatal early life BSp treatment has moderate effects, while adult BSp treatments have shown low activity compared to prenatal/maternal treatment. Furthermore, an BSp diet treatment has been shown to be able to decrease the expression of HDAC1 and increase the levels of acetyl-H3K9 and acetyl-H3K14. These epigenetic modifications were related to a significant up-regulation of tumor suppressor genes such as p53 and p16, and down-regulation of tumor promoting genes such as TERT and c-Myc [141].

In the case of human cervical cancer cells, SFN reduced the expression and activity of DNMT3B and HDAC1 in HeLa cell line. These epigenetic modifications were correlated with a significant reactivation of the tumor suppressor genes RARβ, CDH1, DAPK1, and GSTP1 in HeLa cells [135]. In addition, SFN alone and in combination with EGCG decreased the cell viability of both ovarian cancer cell lines, paclitaxel-sensitive (SKOV3-ip1) and-resistant (SKOV3TR-ip2) cells. In fact, SFN down-regulates the expression of DNMT1, hTERT, and Bcl-2 and up-regulates PARP cleavage and phosphorylated H2AX [132]. These relevant results show the importance of SFN in epigenetic regulation, and therefore the regular consumption of products rich in SFN like broccoli should be present in our eating habits.

## 5. Quinones as Epi-Drugs

**Figure 8:**
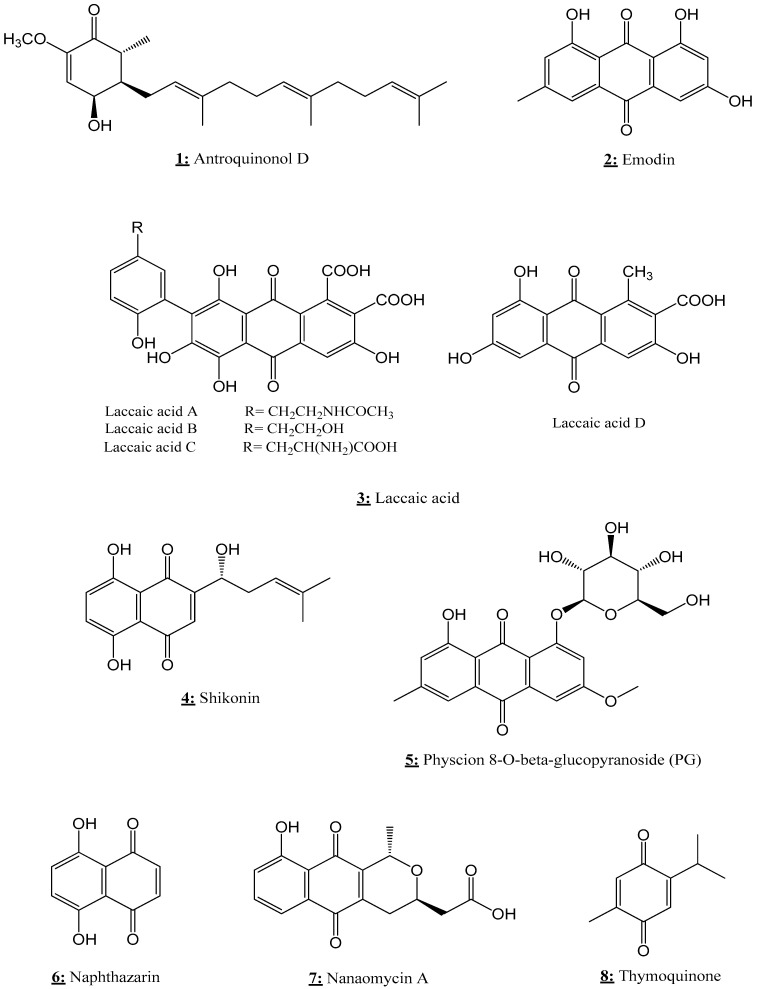
Chemical structures of quinones with anticancer effects via epigenetic targets.

Different compounds from quinone (d-antroquinonol, Emodin, laccaic acid, shikonin, physcion 8-O-β-glucopyranoside, naphthazarin, nanaomycin A, and Thymoquinone) (Figure 8) exhibit anticancer effects by their action on several checkpoints of cancer epigenetic modulators (Table 4).

### 5.1. d-Antroquinonol

d-antroquinonol (3-demethoxyl antroquinonol) (Figure 8), isolated from Antrodia camphorate, has been shown to reduce the growth of breast cancer cells (MCF-7, T-47D, and MDA-MB-231) and inhibit the migration of MDA-MB-231 cell line. At the epigenetic scale, d-antroquinonol induced an important decline of DNMT1 expression and activity, which was correlated with a significant activation of many tumor suppressor genes, specially *FANCC*, *CACNA1A*, *CDH15*, *ASB9*, and *COL4A2*, at both mRNA and protein levels in MDA-MB-231 cells [160,162]. In lung cancer, this natural substance showed cytotoxic activity and inhibited cell (CL1-5 cells) migration capacity. It has also been shown to be able to enhance the expression of cyclin D2 (CCND2) tumor suppressor gene in CL1-5 cells [161].

### 5.2. Emodin

Emodin is a natural compound produced by many medicinal plants that was reported as an epigenetic modulator in several types of cancer (Figure 8). In fact, emodin significantly inhibited the cell growth of four human bladder urothelial cell carcinoma lines (MBT2, T24, TSGH8301, and J82). This cytotoxicity was directly related to the ability of emodin to induce epigenetic modifications. In fact, emodin enhances the expression of H3K27me3 and decreases the expression of pH3Ser10 on the promoter region of many repressed genes. These epigenetic modifications induced by emodin have led to a significant repression of the expression of many oncogenic genes involved in proliferation, inflammation, and carcinogenesis. Among the main repressed genes in bladder cancer, there are fatty acid binding protein 4 (FABP4) and fibroblast growth factor binding protein 1 (HBP17), RGS4, tissue inhibitor of metalloproteinase 3 (TIMP3), WNT5b, URB, and collagen type VIII, alpha 1 (COL8A1) [163].

In lymphoma, emodin showed an anticancer activity against Raji cells by reducing cell viability and increasing apoptosis, with significant up-regulation of caspase-3, caspase-9, and poly (ADP-ribose) polymerase. Emodin has been found able to induce many epigenetic modifications, mainly the increase in DNMT3A and the decrease in UHRF1 expression [167]. UHRF1 (Ubiquitin-like containing PHD and Ring Finger 1) is an oncogenic factor over-expressed in different cancer cells and is known by its potent effect in silencing tumor suppressor genes in cancer cells [175,181].

Additionally, emodin reduces the expression of the anti-apoptotic *ΔNp73* gene, which leads to an increase in the *TAp73*/*ΔNp73* ratio and subsequently to an increase in the pro-apoptotic *TAp73* activity [160,167,168,169,170,171,172,173,174,175,176,177,178,179,180,181,182]. In contrast, other studies reported that emodin was a potent inhibitor of DNMTs [164,165,166].

It has also shown great results in inhibiting cell growth and enhancing demethylation in the pancreatic cancer cell line Panc-1 by reducing the expression of DNMT1 and DNMT3a. Emodin-induced demethylation processes were correlated with a significant up-regulation of three tumor-suppressor genes (*P16*, *RASSF1A*, and *ppENK*) [164,166]. These epigenetic modifications in pancreatic cancer cells have been found highly efficient when emodin was used in combination with the demethylating agent 5-Aza-CdR [166].

In an in vivo study on golden Syrian hamsters, emodin played a key role in the restoration of the epigenetic disorder induced by DMBA (7,12-dimethylbenz[a]anthracene), known as a laboratory tumor initiator. DMBA induced the over-expression of DNMT1, DNMT3a, and DNMT3b with increased levels of p-Akt, p-ERK and p-P38 MAPK, which directly led to oral carcinoma development in treated hamsters. However, administration of emodin to animals treated with DMBA restored normal expression levels of all previous altered genes [165].

On the other hand, emodin could influence the telomerase activity in breast cancer cells. It induced a considerable decrease in c-myc and increased expression of E2F1 resulting in down-regulation of hTERT expression and activity in three breast cancer cell lines (MDA-MB-453, MDA-MB-231, and MCF-7). In addition, the decline of hTERT was also mediated by the stabilization of G-quadruplex structure by emodin [168].

### 5.3. Laccaic Acid

Laccaic acid (Figure 8) could produce epigenetic modifications in colorectal cancer, primarily downregulating DNMT1 and HDAC1 expression. These modifications lead to an increase in cell death by apoptosis and to decrease the cell viability of HT29 cell line in vitro as well as a potent antitumor activity in vivo. LA showed a synergistic effect when used in combination with PEITC [129].

### 5.4. Shikonin

Shikonin (Figure 8) showed great potential as DNMT1 inhibitor. In thyroid cancer, the ihibition of DNMT1 by shikonin resulted in the up-regulation of PTEN expression, resulting in inhibition of TPC-1 cell growth, migration, and invasion [173]. While in human breast cancer (MCF-7) and cervical carcinoma (HeLa) cells, the decline in DNMT1 expression and enhancement of apoptosis by shikonin were correlated with the enhancement of caspase-3, PARP cleavage and p73 expression, and decreased BCL-2 expression with activation of the p16^INK4A^ tumor suppressor gene [172].

### 5.5. Physcion 8-O-β-Glucopyranoside

Physcion 8-O-β-glucopyranoside (PG) (Figure 8) induced a significant attenuation of the invasiveness of breast cancer cells (MDA-MB-231) and Human hepatocellular carcinoma (HepG2). This effect has been found to be related to the ability of PG to suppress EMT via AMPK activation, and to the decline in expression of DNMT1 and Sp1 [169,170]. The PG-induced metastasis-suppressing effect has been confirmed in vivo in the case of breast cancer [170]. Moreover, PG inhibits the growth of testicular germ cell tumors by declining cell proliferation, enhancing apoptosis, and inducing cell cycle arrest in NTERA2 and NCCIT cell lines. On the other hand, the antitumor potential of PG against testicular germ cell tumors was mainly mediated by reactivation of miR-199a by PG [171]. The down-regulation of miR-199a in cancer cells has been found involved in tumor growth and angiogenesis, while reactivation of this repressed-microRNA is considered a potent approach to decline tumor angiogenesis and development [171].

### 5.6. Naphthazarin

Naphthazarin (Naph) (DHNQ, 5,8-dihydroxy-l,4-naphthoquinone) (Figure 8), a natural compound known by its diverse activity such as anti-inflammatory, antioxidant, antibacterial and antitumor, was found able to induce cell cycle arrest and apoptosis in MCF-7 cells via an epigenetic process. In fact, naphthazarin decreases the expression of two epigenetic marks DNMT1 and HDAC1, resulting in the up-regulation of p21 cell cycle inhibitor. Moreover, naphthazarin decreased the expression of the oncogenic factor UHRF1. These naphtazarin-induced modifications were more potent when exposed to ionizing radiation [111].

### 5.7. Nanaomycin A

Nanaomycin A (Figure 8), a quinone antibiotic isolated from Streptomyces, showed cytotoxic activity against three different human tumor cell lines; HCT116 (colon cancer), A549 (lung cancer), and HL60 (tumor cells in bone marrow). This compound induced a significant decline of the genomic methylation levels in the three cell lines. Nanaomycin A was defined as a distinct selective inhibitor of the epigenetic mark DNMT3B activity. In A549 cells, this molecule has been found able to restore the activity of many hypermethylated tumor suppressor gene such as RASSF1A that undergo significant demethylation and therefore subsequent up-regulation upon nanaomycin A treatment [174].

### 5.8. Thymoquinone

Thymoquinone (TQ) (Figure 8) is the most abundant constituent of the Nigella sativa black seeds that is known by its potent and various biological activities such as anti-oxidant, anti-inflammatory and anti-tumor properties with a noticeable health benefits like gastroprotective and hepatoprotective effects [183]. TQ was reported as a potent inhibitor of cancer growth and metastasis in different types of cancer. Especially, this compound has a strong anti-leukemia activity in vitro and in vivo through epigenetic pathways. In fact, TQ has been shown to be an effective inhibitor of many epigenetic marks, especially UHRF1, DNMT1, and HDAC1. Several investigations showed that TQ could induce apoptosis and cell cycle arrest in lymphoblastic leukemia Jurkat cells via the down-regulation of UHRF1 (an oncogenic factor and silenced of tumor suppressor genes). The mechanism of UHRF1 repression has been found linked in part to an up-regulation in cleaved caspase-3 and p73 expression [175,179]. This hypothesis was confirmed by the knockdown of p73 expression that results in the restoration of UHRF1 expression and the inhibition of TQ-induced apoptosis [175]. According to Abusnina et al. [176], the repression of UHRF1 by TQ is mediated via the down-regulation of PDE1A (Phosphodiesterase 1A) in lymphoblastic leukemia Jurkat cells. In fact, TQ has been found able to decline the expression of PDE1A, which leads to the up-regulation of p73 and a subsequent repression of UHRF1. The repression of UHRF1 via the suppression of PDE1A was proven to induce re-expression of PDE1A, which was correlated with a decline of p73 and a reactivation of UHRF1 expression [176]. Additionally, UHRF1 repression could be via its auto-ubiquitination through UHRF1-RING domain upon TQ treatment. TQ-induced UHRF1 ubiquitination was found due to the significant ability of TQ to decrease the expression of HAUSP (herpes virus-associated ubiquitin-specific protease) resulting in UHRF1 down-regulation [175,179]. TQ also exhibits a distinct inhibitory effect on DNMT1 expression in leukemia cells. TQ treatment caused an inhibition of DNMT1 expression at both mRNA and protein levels via the interruption of the Sp1/NF-κB complex in the DNMT1 gene promoter, leading to DNA demethylation [178]. Consequently, all of these TQ-induced epigenetic modifications were associated with the reactivation of many tumor suppressor genes found silenced in cancer cells, such as DDIT3, DLC1, CYP1B1, FOXO6, PPARG, SALL4, ST7, and TET2 [180]. On the other hand, TQ could also inhibit cancer cell migration and invasion. The inhibition of cancer metastases by TQ occurred due to its ability to repress EMT-promoting proteins. In fact, TQ down-regulates the expression of TWIST1, an EMT-promoting transcription factor. TQ treatment also decreased the expression of N-Cadherin, an up-regulator of TWIST1, and increased the expression of E-Cadherin, a suppressor of TWIST1, resulting in reduced cell migration and invasion [177].

## 6. Conclusions

Here, we have highlighted the role of some natural bioactive compounds as epidrugs against cancer diseases. Alkaloids, terpenoids, quinones, and isothiocyanates have been shown to exhibit remarkable effects on epigenetic modifications in human cancer cell lines. Their action involves different mechanisms including the inhibition and/or DNA methylation and histone modifications. Indeed, the reversibility of epigenetic modifications gives the opportunity biochemical interventions via inhibition and/or activation of these modulators, and therefore the possibility to screen epidrugs against cancer, as has been since demonstrated with 5-azacytidine. However, studies highlighted here about natural compounds as epidrugs were almost carried out via in vitro and in vivo approaches and therefore their pharmaceutical applications need further investigations in clinical trials. Indeed, the toxicity of these compounds should be explored to validate their safety, and their pharmacokinetic selectivity should be also investigated to avoid more of their side effects as well. Moreover, the actions of these compounds found in medicinal plants can play an important role as chemoprevention agents because of their use with medicinal plants.

## Figures and Tables

**Table 1:** Alkaloids as epi-drugs against cancer.

Bioactive Molecules	Origin	Cell Lines/Tissues	Experimental Methods	Key Results	References
Berberine (BBR)	Purchased	Multiple myeloma cell line U266	Gene expression microarray Epigenetic chromatin modification enzymes PCR array Gene ontology and KEGG pathway analysis RT-PCR Bisulfite sequencing PCR (BSP) analysis Western blot analysis	Repressed the expression of DNMT1 and DNMT3B, which triggers hypomethylation of TP53 by changing the DNA methylation level and the alteration of p53 dependent signal pathway in U266 cells.	[10]
	Purchased	Multiple myeloma cell line U266 HL-60/ADR and KG1-α cell lines	Reverse docking study Gene expression microarray Epigenetic chromatin modification enzymes PCR array Growth inhibition and apoptosis assay RT-PCR Western blot analysis	Lysine-N-methyltransferase was the putative target of BBR. Affected the enzymes involved in histone acetylation and methylation. Induction of cytotoxicity and apoptosis in HL-60/ADR and KG1-α cells. Up-regulation of histone acetyltransferase CREBBP and EP300, histone deacetylase SIRT3, histone demethylase KDM6A as well as histone methyltransferase SETD7. Down-regulation of histone acetyltransferase HDAC8, histone methyltransferase WHSC1I, WHSC1II, and SMYD3.	[11]
	Purchased	Cultured colon tissues from neonatal rats	Malignancy was induced by TGF-β1 in vitro Hematoxylin and eosin staining Immunohistochemistry RT-PCR	After treatment with BBR and evodiamine for 24 h, increased expression of DNMT1, DNMT3A, DNMT3B and miR-152, miR-429, miR-29a was noted, respectively.	[12]
	Purchased	Human non-small cell lung cancer (NSCLC) cells (A549 and H1975)	Cell viability assay Cell proliferation assay qRT-PCR Western blot analysis	Inhibited growth, migration, invasion, and induced cell cycle arrest in lung cancer cells. Inhibited DNMT1 mRNA, protein and promoter activity. Reduced 3-phos-phoinositide-dependent protein kinase-1 (PDPK1) and transcription factor SP1 protein expressions. The combination with metformin, enhanced the effects of BBR on cell growth, protein levels of SP1, PDPK1, and DNMT1.	[13]
	Purchased	C17.2 cells, a cell line from the mouse neuron stem cells	Cytotoxicity assay RNA extraction and quantitative RT-PCR Genomic DNA isolation and methylation detection Global DNA methylation detection Western blot analysis	Regulated the expression of peroxisome proliferator-activated receptor (PPARγ) in a specific way upon ischemia-reperfusion injury. Enhanced the PPARγ expression during cerebral ischemia-reperfusion. Reduced the global methylation, declined the expressions of DNMT1 and DNMT3a in the ischemia-reperfusion and reduced the methylation of PPARγ promoter region.	[14]
	Not reported	Multiple myeloma (MM) cells	Cell viability assay Proteasome activity assay and quantitative RT-PCR Western blot analysis SPR-LC-MS/MS approach	Killed MM cells in vitro and prolonged the survival of mice bearing MM xenografts in vivo. UHRF1 (ubiquitin-like with PHD and RING Finger domains 1) was the potential target of BBR. BBR may directly bind to the tandem Tudor domain and plant homeodomain (TTD-PHD domain) to induce its degradation via the ubiquitin–proteasome system, thereby up-regulating several tumor suppressor genes and impeding cell growth both in vitro and in vivo.	[15]
Evodiamine	Purchased	Cultured colon tissues from neonatal rats	Malignancy was induced by TGF-β1 in vitro Hematoxylin and eosin staining Immunohistochemistry RT-PCR	After treatment with evodiamine and BBR for 24 h, increased the expression of DNMT1, DNMT3A, DNMT3B, and miR-152, miR-429, miR-29a was noted, respectively.	[12]
3,3′-Diindolylmethane (DIM)	Not reported	Prostate cancer cells (LnCAP and PC3)	ChIP assay Sample preparation for DNA methylation array DNA methylation array data analysis Validation of DNA methylation data Gene expression analyses	DIM + SFN decreased DNMT gene expression and caused distinct DNA methylation profile alterations depending on prostate cell line, but shared similar gene targets within a single cell line. DIM + SFN reversed cancer-associated DNA methylation alterations in LnCAP cells.	[16]
Harmalin	Not reported	NB4 cell line	Cytotoxicity assay Methylation specific PCR RT-PCR	After 48 h, harmalin showed a dose and time-dependent anti-proliferative properties on the NB4 cell line. Harmalin (15 μg/mL) reduced gene expression of DNMT1, induced hypomethylation of P15 gene promoter and increased P15 gene expression in NB4 cell line.	[17]
Harmine	Not reported	HL60 Leukemia cell line	Cell viability assay Quantitative real-time PCR	Suppressed cell proliferation in all concentrations. Reduced cell proliferation. Up-regulated the DAPK expression.	[18]
	Purchased	Human promyelocytic NB4 cell line	Cell proliferation and cell cycle analysis Real-time PCR MSP analysis	Reduced cell proliferation in the NB4 cell line in a time and dose-dependent manner. Increased the number of cells in the G1 phase of the cell cycle. Suppressed the DNMT1 gene in the NB4 cell line. Down-regulated the DNMT1-induced p15 tumor suppressor, promoting hypomethylation and reactivation	[19]
Indicaxanthin (Ind)	Not reported	Colorectal cancer cell lines (CACO2, LOVO1, DLD1, HT29, and HCT116)	MTT assay Quantitative PCR approach Gene-specific methylation analysis Quantification of DNMT and demethylase gene expression DNMT Activity Assay In silico molecular modelling approach	Exhibited anti-proliferative activity in all cell lines, except HT29. Induced demethylation in the promoters of some methylation-silenced onco-suppressor genes involved in colorectal carcinogenesis (p16^INK4a^, GATA4, and ESR1), and left unchanged others which were basally hypermethylated (SFRP1 and HPP1). Increased DNMT gene expression. Inhibited DNMT activity. Increased the expression of genes involved in DNA demethylation. Stable binding of Ind at the DNMT1 catalytic site.	[20]
Isofistularin-3 (Iso-3)	*Aplysina aerophoba*	RAJI, U-937, JURKAT, K-562, HL-60, MEG-01, and PC-3 cells	In vitro DNMT and HDAC activity assays Docking analysis mRNA expression analysis CpG methylation analysis Cell proliferation and viability assays (RAJI cells) Western blot analysis	Inhibited DNMT1 in vitro by binding to the DNA interacting pocket of the enzyme. Modified the aryl hydrocarbon receptor (AHR) promoter methylation and increased the AHR expression in RAJI cells. Induced growth arrest of cancer cells in G0/G1 concomitant with increased p21 and p27 expression and reduced cyclin E1, PCNA, and c-myc levels. Induction of morphological changes and autophagy in RAJI cells.	[21]
Betanin	Not reported	Epithelial breast cancer MCF7 cell line	Determination of the activity of DNMTs DNA methylation analysis Real-time PCR Western blot analysis	Inhibited the DNMT activity. No effect on the methylation pattern or the expression of *RASSF1A*, *GSTP1,* or *HIN-1* in MCF7 cells. No effect on the global methylation of histone H3, No effect on DNMT1 transcription or on DNMT1 protein level.	[22]
Mahanine	*Murraya koenigii*	PC3, LNCaP, A431, A549, ASPC-1, HT-29, MCF7, and SKOV-3 cells	Cell culture Western blot analysis DNMT activity assay RT-PCR	Induced the expression of an epigenetically silenced gene *RASSF1A* in human prostate and various other cancer cells. Reduced cyclin D1 but not other cyclins. Restored the expression of *RASSF1A* by inhibiting DNMT activity in human prostate cancer cells (LnCAP and PC3).	[23]
	*Murraya koenigii*	Human prostate cancer cell lines (PC3 and LnCaP)	Western blot analysis RASSF1A promoter methylation assay RT-PCR	Restored the expression of *RASSF1A* by inducing the demethylation of its promoter in prostate cancer cells. Induced the degradation of DNMT1 and DNMT3B, but not DNMT3A, via the ubiquitin-proteasome pathway. Decreased the phospho-Akt levels and disrupted the interaction of Akt with DNMT1 and DNMT3B.	[24]
Nicotinamide (NA)	Not reported	Human breast cancer cell line MCF-7	Cell viability assay Apoptosis assay Western blot analysis RT-PCR	Combination therapy with nicotinamide and valproate inhibited the viability of MCF-7 cells, decreased cell activity, suppressed cell proliferation, and up-regulated p16 and p21. Detected high levels of acetylated histone H3 in nicotinamide- and valproate-treated cells.	[25]
	Purchased	Placenta, fetal liver, and whole brain samples	Genomic DNA methylation assay Measurement of uracil levels in DNA RT-PCR	Decreased placental and fetal hepatic genomic DNA methylation and genomic uracil contents (a factor modifying DNA for diversity) in the placenta and fetal liver and brain. Induced tissue-specific alterations in the mRNA expression of the genes encoding nicotinamide N-methyltransferase, DNMT1, catalase and tumor protein p53 in the placenta and fetal liver. Increased fetal hepatic α-fetoprotein mRNA level (at 4 g/kg).	[26]
	Purchased	Excised skin from the painted area (female mice)	Quantitative real-time reverse transcription PCR Bisulfite modification of DNA and methylation specific PCR (MS-PCR) RT-PCR Western blot analysis	Prevented tumor development but protection was greatly improved when combined with butyric acid (BA) and calcium glucarate (CAG). Downregulated the miR-203 levels at 16 weeks. Up-regulated the histone deacetylases (HDAC), DNMT, and promoter methylation of miR-203 at 4 or 16 weeks. Prevented altered gene expression (after 16 weeks), while co-administration with BA and CAG had a more pronounced effect than that of the individual compound, by regulating miR-203 status through epigenetic or biogenetic modulations.	[27]
Psammaplin	*Pseudoceratina purpurea*	Not reported	In vitro cell proliferation assay HDAC enzyme inhibition assay	Psammaplin A (4) and psammaplin F (10) are potent HDAC inhibitors with mild cytotoxicity. Psammaplin A (4) and psammaplin G (11) are potent DNMT inhibitors.	[28]
	*Jaspis* sp. and *Poecillastra wondoensis*	Human endometrial Ishikawa cancer cell line	Cell proliferation RT-PCR Western blot analysis	Psammaplin A (PsA) inhibited the proliferation of endometrial cancer cells in a dose-dependent manner. Induced accumulation of acetylated histones and reduced HDAC level. Up-regulated the expression of cyclin-dependent kinase (CDK) inhibitor p21^WAF1^. Down-regulated the expression of pRb, cyclins, and CDKs, which lead to induce cell cycle arrest. Increased the cellular proportion in the G1 phage and G2/M phage	[29]
	*Pseudoceratina purpurea*	Human cancer cell lines	HDAC assay DNMT assay Immunoblotting	PsA (11c) had a highly potent effect against HDAC1 in vitro (IC_50_ = 0.9 nM) PsA had high isoform selectivity, being 360- fold selective for HDAC1 over HDAC6 and more than 1000-fold less potent against HDAC7 and HDAC8 PsA showed significant cytotoxicity in A549, MCF7, and W138 cells Up-regulated the histone acetylation	[30]
Reserpine	*Rauvolfia verticillata* (Lour.) Baill.	Human hepatocellular HepG2-C8 cell line Mouse skin epidermal JB6 P+ cells	Cell viability assay RNA isolation and quantitative RT-PCR Western blot analysis Methylation DNA immunoprecipitation assay	Decreased the TPA (12-O-tetradecanoylphorbol-13-acetate)-induced colony formation of JB6 cells in a dose-dependent manner Demethylation effect on the first 15 CpGs of the Nrf2 promoter in JB6 P+ cells. Reduced the mRNA and protein expression of DNMT1, DNMT3a, and DNMT3b. Induced Nrf2 expression via an epigenetic pathway in skin epidermal JB6 P+ cells and enhancing the protective antioxidant activity.	[31]
Solamargine	Purchased	Human cancer lines H1650, H1975, PC9, A549, and H1299	Cell viability assay Cell cycle analysis Western blot analysis Transient transfection assay	Inhibited growth in multiple lung cancer cell lines and induced cell growth arrest in H1299 NSCLC cells. Inhibited protein expression of DNMT1 through activation of ERK1/2 signaling. Reduced PGE2 receptor EP4 protein. Inhibited c-Jun protein through inhibition of DNMT1 expression.	[32]
Vincristine	Not reported	A colon cancer cell line (DLD-1) and a normal colon cell line (CCD18Co)	Quantitative methylation-specific PCR Sodium bisulfite DNA modification Quantitative RT-PCR mRNA extraction and cDNA synthesis	Demethylated the runt-related transcription factor-3 (RUNX3) in DLD-1 cells. Restored the expression of RUNX3 mRNA in DLD- 1 cells. Detected the hypermethylation of RUNX3 in 70 out of 105 colorectal carcinomas (66.7%). Reduced the expression of RUNX3 mRNA in 68 out of 105 colorectal cancer tissues (64.8%).	[33]
Trichostatin A (TSA)	Not reported	Jurkat T leukemia cells clone E6-1	Real-time quantitative PCR Western blot analysis Measurement of DNMT1 mRNA stability	Down-regulated the DNMT1 mRNA and protein expression in Jurkat T leukemia cells clone E6-1. Decreased the DNMT1 mRNA stability.	[34]
	Purchased	Human melanoma cell line A2058	Detection of S1P (sphingosine-1-phosphate) receptor mRNA expression RT-PCR analysis Cell motility assay Analysis of signaling proteins Analysis of DNA methylation for a putative S1P_1_ promoter Cell viability assay	TSA + Aza-dC induced epigenetic regulation of S1P receptors in human melanoma cells, switching S1P from motility inhibitor to stimulator. Increased expression of S1P_1_ and S1P_3_, associated with S1P-induced chemotaxis, and decreased expression of S1P_2_, associated with motility inhibition.	[35]
	Purchased	T24 and MDA-MB-231 cells	RT-PCR Western blot analysis ChIP assay	Increased the histone acetylation, associated with a significant decrease in global methylation. Induced the histone acetylation, demethylation, and expression of the methylated *E-CADHERIN* and *RARβ2* genes. Induction of acetylation and demethylation by TSA showed some gene selectivity.	[36]
	Purchased	The pancreatic (PANC-1, CAPAN-1, and CAPAN-2) and gastric (KATO-III) epithelial cancer cell lines	RNA extraction, RT-PCR, and quantitative real-time PCR Western blot analysis ChIP assay siRNA assay	TSA + 5-aza restored the *MUC4* expression in a cell-specific manner. DNMT3A, DNMT3B, HDAC1, and HDAC3 were directly involved in *MUC4* silencing by binding to its 5′-UTR in a cell-specific manner. Inhibited the histone deacetylation, associated with strong *MUC4* repression in high-expressing cells.	[37]
	Not reported	HCT116 human colon cancer cell line	Quantification of *hTERT* mRNA Bisulfite modification and PCR-directed sequencing Flow cytometry assay Western blot analysis ChIP assay	Induced the demethylation of site-specific CpGs on the promoter of *hTERT*, which was caused by the down-regulation of DNMT1. Promoted the CTCF binding on *hTERT* promoter, leading to repression of *hTERT*.	[38]
	Not reported	Colon cancer cell lines SW480, DLD-1, HCT15, HT29, RKO, and SW48	Quantitative real-time PCR Western blotting analysis *DKK1* Overexpression	Increased the mRNA expression of *DKK1* in a dose-dependent manner.	[39]
	Purchased	CA46 human Burkitt lymphoma cell line	Cell proliferation analysis Cell cycle analysis Nested methylation-sensitive PCR Real-time PCR Western blot analysis	TSA inhibited CA46 cell proliferation. TSA (15 ng/mL) + EGCG (6 μg/mL) reduced CA46 cell proliferation from 24 to 96 h. TSA + EGCG decreased p16^INK4A^ gene methylation, which coincided with increased p16^INK4A^ mRNA and protein expression. TSA + EGCG reactivated p16^INK4A^ gene expression in part through reducing promoter methylation, which may decrease CA46 cell proliferation.	[40]
	Purchased	Breast cancer cell lines (MCF-7, MDA-MB-231, and MDA-MB-157) Normal human mammary epithelial cells (HMECs) Two mouse models	MTT assay RT-PCR assay Western blot analysis ChIP assay	Reactivated the estrogen receptor-α (*ERα*) expression. TSA + GEN enhanced the reactivation of *ERα* expression in MDA-MB-231 cells. TSA + GEN re-sensitized ERα-negative breast cancer cells to E2 and TAM. TSA + GEN induced histone modification changes in the *ERα* promoter. Reduced the HDACs activity (alone or in combination with GEN). No effect on the DNMTs activity.	[41]
	Purchased	Ovarian cancer SKOV3 cell line	Cell viability assay Tumorigenicity of SKOV3 cells Cell migration and invasion assay In vivo implantation assay Western blot analysis Histone immunoblots	TSA + decitabine markedly inhibited the activity of DNMTs and HDACs, especially the expression of DNMT3A/3B and HDAC1/2. TSA + decitabine stimulated the acetylation of histone H3 and H4. TSA + decitabine suppressed the expression level of lysine-specific demethylase-1 (LSD1). TSA + decitabine induced the transcription activity marker dimethylated-H3K4. TSA + decitabine suppressed the dimethylated-H3K9. TSA + decitabine suppressed the tumorigenicity and metastasis of SKOV3 cells in xenograft mouse models. TSA + decitabine suppressed migration capacity through the induction of E-cadherin and suppression of N-cadherin. TSA + decitabine suppressed invasion (at least partially) through inhibition of MMP-2 and MMP-9.	[42]
	Purchased	Human HCC HepG2 cells	MTT cell proliferation assay Real-time qRT-PCR Flow cytometric analysis of apoptotic cells	TSA + GEN inhibited cell growth with all concentrations used. Down-regulated the DNMT1 gene expression after 48 h and 72 h, and DNMT3a gene expression only after 72 h. Induced the apoptosis in all treatment groups.	[43]
	Purchased	Hepatocellular carcinoma Hepa 1-6 cell line	MTT assay Flow cytometry assay Real-time RT-PCR analysis	Inhibited the apoptotic effects, and reduced the expression of DNMT1 The relative expression of DNMT1 gene was 0.5 to 0.19	[44]
	Purchased	Hepa 1-6 cells	Cell growth and cell viability assay Cell apoptosis assay qRT-PCR	Indicated a dose- and time-dependent significant antiproliferative effects (IC_50_~1 μM) Indicated significant apoptotic effects in all different periods. Increased significantly the *ERα* gene expression quantity.	[45]

**Table 2:** Terpenoids as epidrugs against cancer.

Bioactive Molecules	Origin	Experimental Methods	Key Results	References
All-*trans* retinoic acid (ATRA)	Purchased	Human breast cancer cell lines MCF-7, MDA-MB-231, SK-BR3, MDA-MB-453, and HS578T RNA Isolation, RT-PCR, and real-time PCR quantification	ATRA + valproic acid + Aza-dC restored the expression of RARb2 in MCF-7 breast cancer cells. ATRA + valproic acid + Aza-dC restored and enhanced the RARb2 expression in breast cancer cells. ATRA + valproic acid + Aza-dC inhibited the breast cancer cell proliferation.	[84]
	Purchased	MCF-7 and MDA-MB-231 cells Cell culture, proliferation, and viability assay RNA and DNA extraction Methylation analysis of *RARbeta2* promoter Quantitative analysis of *RARbeta2* expression on mRNA level	Methylated, partially, the *RARbeta2* promoter in the tested fragment in MCF-7 cells. Inhibited the promoter methylation and increased the expression of *RARbeta2* in MCF-7 cells. Improved the action of 2CdA (2-chloro-2′-deoxyadenosine) and F-ara-A (9-beta-d-arabinosyl-2-fluoroadenine) on *RARbeta2* methylation and/or expression. Induced the *RARbeta2* expression in MDA-MB-231 cells without any notable effects in combined treatment.	[85]
	Not reported	HepG2 cell line RNA interference Western blot analysis ChIP assay DNMT activity assay MSP	Induced the promoter hypomethylation of p16 and p21 via the down-regulation of DNMT1, 3a, and 3b to facilitate binding of Ets1/2 to the p16 promoter and p53 to the p21 promoter, resulting in the upregulation of their expression and the subsequent induction of cellular senescence in HepG2 cells. Up-regulated the expression of RAR-β2 (retinoic acid receptor-β2) via promoter hypomethylation.	[86]
	Purchased	AR^−^ human prostate cancer cells RT-PCR and real-time PCR Western blot analysis Co-immunoprecipitation (CoIP) and ChIP assays Cell proliferation assays Extraction of genomic DNA and analysis of DNA methylation	Induced the growth arrest and increased the HOXB13 expression in AR^−^ prostate cancer cells Impaired the EZH2 and DNMT3b expression and weakened their interactions with HOXB13 promoter. Reduced the methylation level of the HOXB13 promoter.	[87]
	Purchased	MCF-7 and MDA-MB-231 cells Cytotoxicity assay RNA extraction and complementary DNA synthesis Real-time PCR Methylation-sensitive restriction analysis	Reduced the *PTEN* promoter methylation in MCF-7 cells.	[88]
	Purchased	A549, HepG2, Hep3B, HCT116, and MCF-7 cells Cell viability analysis Cell cycle analysis DNMT activity assay Luciferase reporter assay Western blot analysis Immunoprecipitation (IP) assay MSP RT-PCR and qRT-PCR	Induced the apoptosis in p53-positive human hepatoma cells. Activated the p53-dependent apoptotic pathways in human hepatoma cells. Up-regulated the p53 levels via activation of the p14-MDM2-p53 pathway. Activated the p14 expression via promoter hypomethylation. Abolished the potential of p53 to inhibit p14 expression via DNA methylation. Down-regulated the protein levels of MDM2 via ubiquitin-dependent proteasomal degradation. Induced the apoptosis in other p53-positive human cancer cells via the activation of the p14-MDM2-p53 pathway.	[89]
	Purchased	NSCLC cell lines A549, NCI-H460, and HCC827 Growth inhibition and measurement of apoptosis Western blot analysis	ATRA + panobinostat had additive and synergistic effects, respectively, on growth inhibition and differentiation, with almost no cytotoxicity. ATRA + panobinostat had an effect on histone acetylation. ATRA + panobinostat additively decreased the expression of phospho-ERK and phospho-AKT, whereas p53 and p21^CIP1/WAF1^ proteins were both induced.	[90]
	Purchased	Human erythroleukemic cell line K562 Cell growth and viability assay Methylation-sensitive restriction analysis (MSRA) Measuring the amount of DNMT1 protein	ATRA + clofarabine inhibited cell growth and induced caspase-3-dependent apoptosis. ATRA + clofarabine down-regulated the DNMT1 and up-regulated the CDKN1A, with a concomitant enhanced decrease in the DNMT1 protein level. ATRA + clofarabine induced a concurrent methylation-mediated *RARB* and *PTEN* reactivation,	[91,92]
	Purchased	Human acute myeloid leukemia KG-1 and MOLM-13 cells RNA extraction and qRT-PCR MSP and bisulfite genomic sequencing (BGS) RNA sequencing Western blot analysis Assays of cell growth inhibition, apoptosis and cell cycle	ATRA + DAC induced the growth inhibition, cell cycle arrest, and apoptosis of AML (acute myeloid leukemia) cells. ATRA + DAC inhibited DNMT1, activated miR-34a via promoter hypomethylation, down-regulated its target MYCN, and thus exerted a synergistic antineoplastic effect.	[93]
Boswellic acid	Purchased	Human CRC cell lines RKO, SW48 and SW480 MTT assay BrdU cell proliferation assay Immunofluorescence DNA and RNA extraction Genome-wide DNA promoter methylation and gene expression analyses MSP and quantitative real-time RT-PCR DNMT inhibition assay	Inhibited the cell viability and proliferation. Induced the apoptosis in CRC cells. Induced the demethylation of several CpG loci in CRC cells. Demethylated and up-regulated a subset of genes Inhibited the DNMT activity.	[94]
Corosolic acid	Purchased	TRAMP-C1 cells Cell viability assay Colony formation assay RNA extraction and quantitative real-time PCR DNA extraction and BGS MeDIP analysis ChIP assay Western blot analysis HDAC and DNMT activity	Decreased cell viability in a time- and dose-dependent manner. Increased the mRNA and protein expression levels of Nrf2 and its downstream genes. Decreased CpG methylation in the promoter region of Nrf2. Increased the acetylation of histone H3 lysine 27 (H3K27ac). Decreased the trimethylation of histone H3 lysine 27 (H3K27Me3) in the promoter region of Nrf2. Down-regulated the expressions and activities of epigenetically modifying enzymes in TRAMP-C1 cells.	[95]
	Purchased	Mouse epidermal JB6 P+ cells Cell viability assay RNA-seq sample preparation DNA methyl-seq sample preparation Quantitative PCR	Down-regulated the small proline-rich protein (Sprr2h). Reversed the differentially methylated regions (DMRs) in genes like Dusp22 (dual specificity protein phosphatase 22) and Rassf (tumor suppressor gene family, Ras association domain family) in JB6 P+ cells. Modulated the CDK1 (Cyclin-dependent kinases 1) and RASSF2 (Ras association domain family member 2) genes.	[96]
Cucurbitacin B	*L. graveolense* Roxb.	Human NSCLC cells Cell proliferation assay Cell cycle distribution and apoptosis analysis RNA extraction and quantitative real-time PCR Western blot analysis HDACs and DNMTs activity assays Immunohistochemical staining	Inhibited cellular proliferation and induced cellular apoptosis. Altered the expression of key tumor-related genes. Altered the expression and activity of epigenetic modulatory enzymes. Altered histone modifications at the p16^INK4A^, *p21^CIP1/WAF1^*, and *hTERT* promoters. Inhibited the NNK-induced lung tumorigenesis. Altered the expressions of epigenetic enzymes and tumor-related genes in NNK-induced lung tissues.	[97]
	*Trichosanthes cucumerina*	Breast cancer cells (MCF-7, MDA-MB-231) and breast epithelial cells (MCF-10A) MTT assay Colony formation assay RNA extraction and real-time RT-PCR Western blot analysis	Inhibited cell growth in breast cancer cells. Up-regulated the DNMT1 and obvious heavy methylation in the promoters of c-Myc, cyclin D1, and survivin, which consequently down-regulated the expression of all these oncogenes.	[98]
Grifolin	Not reported	Nasopharyngeal carcinoma CNE1 and 5–8F, gastric MGC803, breast MCF7 and cervical cancer HeLa cell lines Molecular modeling In vitro kinase assay In vitro and ex vivo pull-down assays Fluorescence quenching assay Immunofluorescence analysis Cell invasion assay Evaluation of anti-metastatic acitivity of grifolin in nude mice	Inhibited the kinase activities of ERK1/2 Suppressed the adhesion, migration, and invasion of high-metastatic cancer cells. Inhibited tumor metastasis in a metastatic mouse model. Decreased phosphorylation of Elk1 at Ser383 and the protein. Down-regulated the mRNA level of DNMT1. Inhibited the transcription activity of Elk1 as well as its binding to the DNMT1 promoter region. Increased the mRNA levels of *TIMP2* and *PTEN*.	[99]
	Not reported	Nasopharyngeal carcinoma (NPC) CNE1, CNE1-LMP1, and C666-1 cells MSP assay DNMT activity measurement Isolation of mitochondrial protein fractions Immunofluorescence analysis	Attenuated the glycolytic flux and recovered mitochondrial OXPHOS function by inhibiting DNMT1 expression and activity as well as its mitochondrial retention in NPC cells.	[100]
Hinokitiol	Purchased	Human colon cancer cell lines (HCT-116 and SW480) Cell viability assay and morphologic RNA extraction and RT-PCR qRT-PCR Genomic DNA extraction Western blot analysis Transient transfection of DNMT1 small RNA interference	Reduced the DNMT1 and ubiquitin-like plant homeodomain and RING finger domain 1 (UHRF1) expression in HCT-116 cells. Increased the expression of TET1. Increased the 5-hydroxymethylcytosine (5hmC) level in both colon cancer cells. Restored the mRNA expression of O6-methylguanine DNA methyltransferase (*MGMT*), carbohydrate sulfotransferase 10 (*CHST10*), and B-cell translocation gene 4 (*BTG4*) concomitant with the reduction of the methylation status in HCT-116 cells.	[101]
Eugenol	Purchased	Human cervical cancer cell line Hela Cytotoxicity analysis Apoptosis analysis mRNA expression analysis Protein extraction Western blot analysis Methylation analysis	Eugenol in combination with EGCG + amrogentin could highly: Inhibit cellular proliferation and colony formation; Induce apoptosis; Down-regulate the cyclinD1 and up-regulate the cell cycle inhibitors LIMD1, RBSP3, and p16 at the G1/S phase of the cell cycle; Induce promoter hypomethylation of LimD1 and P16 genes as a result of the reduced expression of DNMT1.	[102]
Parthenolide (PTL)	Purchased	Western blot analysis Real-time RT-PCR Immunoprecipitation HDAC activity assay ChIP assay siRNA transfection Cytotoxicity assay	Depleted the HDAC1 protein without affecting other class I/II HDACs. Promoted the HDAC1 depletion and cell death through the DNA damage transducer ataxia telangiectasia mutated.	[103]
	Not reported	Leukemia cell lines MV4-11, Kasumi-1 and K562 RT-PCR Western blot analysis Electrophoretic mobility shift assays ChIP assay In vitro fluorescent-based ELISA assay Molecular modeling	Induced global DNA hypomethylation and enhanced histone acetylation in a dose- and time-dependent manner in leukemia cells. Inhibited M. SssI activity with an EC_50_ of 5.0 µM. Depleted the DNMT1 protein at 10 µM. Down-regulated the DNMT1 transcription in a time- and dose-dependent fashion. Covalent binding to the catalytic active site by the Michael addition of the cysteine of DNMT1 to the γ-methylene lactone in parthenolide. Increased the histone acetylation without alteration of the enzyme level. Up-regulated the p21.	[104]
	Purchased	MV4-11, MCF-7, and Kasumi-1 cells Human DNMT1 homology modeling Cytoxicity, cell cycle and apoptosis analysis Quantification of global DNA methylation Immunoblotting ChIP assay Xenograft animal model study Real time RT-PCR	Inhibited the DNMT1 (IC_50_=3.5 μM), possibly through alkylation of the proximal thiolate of Cys^1226^ of the catalytic domain by its γ-methylene lactone. Down-regulated the DNMT1 expression possibly associated with its SubG_1_ cell-cycle arrest or the interruption of transcriptional factor Sp1 binding to the promoter of DNMT1. Reactivated the tumor suppressor *HIN-1* gene in vitro, possibly associated with its promoter hypomethylation.	[105]
	Purchased	Acute myeloid leukaemia (AML) cells, including primary AML blasts Methylcellulose colony-forming assays Flow cytometry Western blot analysis NF-κB activity assays	Prevented the HDACi (Histone deacetylase inhibitor)-induced activation of the canonical NF-κB pathway. Potentiated the HDACi-induced apoptosis in various human AML cell lines. Enhanced the HDACi lethality in primary AML blasts. PTL + HDACis induced the apoptosis in MLL-ENL cells displaying leukemia-initiating cell (L-IC) characteristics. PTL + HDACis induced the activation of SAPK/JNK.	[106]
	Not reported	Murine and human epidermal cell lines and JB6 cells Cell growth and cell-cycle analysis Dual luciferase assay for NF-κB transcriptional activity HDAC activity assay Western blot analysis ChIP assay Real-time RT-PCR siRNA transfection Xenograft mouse model	Inhibited the growth of tumor epidermal cells in human and mouse in vitro models. Inhibited tumor promotion in 2D and 3D cultures. Induced S-phase arrest in nonpromoted cells and blocked tumor-promoted cells at S to G2–M phases. Inhibited the tumor promoter–induced NF-κB activity and modulated *p21* and *cyclin D1* NF-κB target genes. Modulated the p65 binding and chromatin structure on *p21* and *cyclin D1* promoters regulating their gene expression. Inhibited the TPA-induced tumor growth in vivo.	[107]
	Purchased	Breast carcinoma MDA-MB231 cells Cell viability assay Analysis of reactive oxygen species (ROS) Measurement of GSH content Measurement of NF-κB activity Western blot analysis	Stimulated the survival pathway Akt/mTOR and the consequent nuclear translocation of Nrf2. PTL + HDACi induced GSH depletion, a fall in ΔΨm, the release of cytochrome c, the activation of caspase 3 and apoptosis. PTL + HDACi maintained the hyperacetylation of both histones H3 and H4 induced by HDACi and the down-regulation of DNMT1 expression induced by PTL. HDACi increased the cytotoxic effect of PTL.	[108]
Ursolic acid	Purchased	Human HCC cells HepG2 Cell viability assay Cell apoptosis assays Western blot analysis Electroporated transfection assays	Inhibited the growth of HCC cells and induced apoptosis in a dose- and time-dependent fashion. Induced phosphorylation of AMPKα and suppressed the protein expression of DNMT1 in a dose-dependent manner. Suppressed the expression of transcription factor Sp1.	[109]
	Purchased	Human NSCLC cells Cell viability assay Cell apoptosis assays Detection of caspase 3/7 activity Western blot analysis Electroporated transfection assays	Inhibited the growth and induced the apoptosis of NSCLC cells in a dose- and time-dependent fashion. Induced the phosphorylation of SAPK/JNK and suppressed the protein expression of DNMT1 and EZH2. Suppressed the expression of the SP1 protein.	[110]
	Purchased	JB6 P+ mouse epidermal cells Cell viability assay Anchorage-independent cell transformation assay RNA extraction and qPCR Western blot analysis DNA isolation and BGS HDAC activity assay	Inhibited the growth of JB6 P+ cells. Inhibited the TPA-induced transformation of JB6 P+ cells. Up-regulated the Nrf2 and its downstream detoxifying/antioxidant target genes. Decreased Nrf2 promoter methylation. Reduced the expression of epigenetic modifying enzymes, including the DNMT1 and DNMT3a and the HDAC1, 2, 3, and 8 (Class I) and HDAC6 and 7 (Class II), and HDAC activity.	[111]
	Purchased	Measurement of mRNA expression in leukocytes qPCR PK/PD model development Male Sprague-Dawley rats	Suppressed iNos expression induced by LPS. Attenuated the induction of the epigenetic markers (DNMT1, DNMT3a, HDAC1, and HDAC3) in leukocytes, mediated by LPS (lipopolysaccharide). Increased the expression levels of Ho1, Nqo1, and Ugt1a1.	[112]
	Purchased	Human myeloma cells (RPMI8226) In vitro gel-based USP7 activity assay Cell apoptosis assays Western blot analysis Molecular docking	IC_50_ = 7.0 ± 1.5 μmol/L Occupied the ubiquitin-binding pocket of USP7, with the 17-carboxyl group and 3-hydroxyl group playing a vital role in the USP7-ursolic acid interaction Interacted with USP7 in RPMI8226 human myeloma cells. Inhibited the proliferation of the myeloma cells (IC_50_ = 6.56 μmol/L), accompanied by reductions in USP7 substrates such as MDM2, UHRF1, and DNMT1.	[113]
Z-Ligustilide (LIG)	Purchased	TRAMP C1 cells MTS Assay RNA isolation and qPCR Preparation of protein lysates and western blot analysis BGS MeDIP analysis In vitro methylation assay	LIG + RAS (Radix *Angelicae Sinensis*) induced the mRNA and protein expression of endogenous Nrf2 and Nrf2 downstream target genes, such as HO-1, NQO1, and UGT1A1. LIG + RAS decreased the level of methylation of the first five CpGs of the Nrf2 promoter. LIG + RAS decreased the relative amount of methylated DNA in the Nrf2 gene promoter region. LIG + RAS inhibited DNMT activity in vitro.	[114]
	Purchased	MDA-MB-231, MDA- MB-453, and HS578t Cell viability assay Colony formation assay Cell apoptosis and cell cycle analysis by flow cytometry Western blot analysis Luciferase assay ChIP assay Immunoprecipitation assay	Restored the growth inhibition of tamoxifen (TAM) on ERα^-^ breast cancer cells. LIG + TAM induced the apoptosis and S and G2/M phases cell cycle arrest. Reactivated the ERα expression and transcriptional activity. Increased the Ace-H3 (lys9/14) enrichment in the ERα promoter. Reduced the enrichment of metastasis-associated protein 1 (MTA1) as well as IFN-γ-inducible protein 16 (IFI16) and HDACs onto the ERα promoter. Down-regulated the MTA1, IFI16, and HDACs, which caused destabilization of the corepressor complex.	[115]
β-elemene	Purchased	Human NSCLC cells Cell viability assay Western blot analysis Treatment with Sp1 small interfering RNAs (siRNAs) Electroporated transfection assays	Inhibited the NSCLC cell growth and increased the phosphorylation of ERK1/2, Akt, and AMPKα. Inhibited the expression of DNMT1. Suppressed the Sp1 protein expression, which was eliminated by either the ERK1/2 or AMPK inhibitor.	[116]
	Purchased	Nasopharyngeal carcinoma (NPC) cells Cell viability assay Cell cycle analysis Western blot analysis Transient transfection assays Xenograft experiments	Reduced the phosphorylation of the signal transducer and the activator of transcription 3 (Stat3) and the protein expressions of DNMT1 and the enhancer of zeste homolog 2 (EZH2). Inhibited tumor growth, thephosphorylation of Stat3, and the expressions of DNMT1 and EZH2 in a mouse xenograft model. Inhibited the NPC cell growth via the inactivation of Stat3 and reduced the DNMT1 and EZH2 expressions	[117]

**Table 3:** Isothiocyanate as epi-drugs against cancers.

Bioactive Molecules	Origin	Experimental Methods	Key Results	References
*Moringa* isothiocyanate	Not reported	Mouse epidermal JB6 P+ cell line Cell viability test: MTS assay RNA-seq DNA SureSelect Methyl-seq	Induced the apoptosis of JB6 P+ cells in a dose- and time-dependent manner. Altered the gene expression profiles in mouse epidermal cells. Affected the canonical signaling pathways. Played a protective role during TPA-induced neoplastic/tumorigenic transformation in JB6 cells. Changed the DNA methylation during TPA-induced neoplastic/tumorigenic transformation in JB6 cells. Reversed the methylation changes in genes (hyper- or hypomethylation) that occur in a response to TPA.	[16]
Phenethyl isothiocyanate (PEITC)	Purchased	PCa LNCaP and PC3 cell lines RNA isolation, miRNA profiling, and qPCR Transfection of has-miR-194-5p mimic and inhibitor Western blot analysis Luciferase reporter activity assay RNA interference	Altered the miRNA expression in PCa cells miR-194 and suppressed PC3 cell invasiveness in vitro. Down-regulated the MMP2 and MMP9 via microRNA-194. Decreased BMP1 expression which decreased cellular MMP levels.	[126]
	Not reported	Colorectal cancer cell lines SW620, SW480, and HCT116 Cell viability analysis Western blot analysis RNA extraction and quantitative real-time PCR Epigenome-wide DNA methylation assays Cell cycle analysis	Induced stable alterations in the expression profile of epigenetic writers/erasers and chromatin-binding of HDACs and Polycomb-group (PcG) proteins. PEITC exposure not only blocked HDAC binding to euchromatin but was also associated with hypomethylation of PcG target genes that are typically hypermethylated in cancer. Induced the expression of pro-apoptotic genes in tumor cells.	[127]
	Purchased	PCa LNCaP cell line Setd7 knockdown in LNCaP cells RNA isolation Oligonucleotide microarray analyses for transcriptome profiling qPCR	Impacted a large set of genes and caused a high fold change. Altered several signaling pathways, in particular inflammation-related TNFR signaling and PTEN/PI3K/AKT signaling.	[128]
	Purchased	Human colon carcinoma cell line HT29 Cell viability assay Cell cycle arrest analysis Sprague Dawley rats Determination of DNMT1 and HDAC1 levels	PEITC + laccaic acid (LA) reduced cell viability with apoptotic cell death. Induced necrotic cell death. PEITC + LA attenuated the number of aberrant crypt foci, DNMT1, and HDAC1 levels (in vivo).	[129]
Sulforaphane (SFN)	Not reported	Breast cancer MCF-7 and MDA-MB-231 cells Total RNA extraction, RT-PCR, and real-time quantitative PCR Western blot analysis HDAC, HAT, and DNMT activity assays Bisulfite sequencing analysis ChIP analysis Apoptosis assay	Inhibited the viability and proliferation of breast cancer cells in vitro. Inhibited the *hTERT* in both breast cancer cells in a dose- and time-dependent manner. Decreased DNMT1 and DNMT3a in breast cancer cells. Increased the level of active chromatin markers acetyl-H3, acetyl-H3K9, and acetyl-H4. Decreased the trimethyl-H3K9 and trimethyl-H3K27 inactive chromatin markers in a dose-dependent manner.	[130]
	Purchased	Human colon adenocarcinoma cell lines Caco-2 and HCT116 Cell proliferation assay Genomic DNA and RNA isolation Quantification of gene-specific CpG island and global (LINE-1) methylation Real-time PCR	No effect on the methylation of CpG islands in ESR1, p16^INK4A^ or of LINE-1, a marker of global genomic methylation. Induced transient changes in DNMT mRNA expression.	[131]
	Purchased	Ovarian cancer cell lines (SKOV3-ip1 and SKOV3TR-ip2 cells) MTT assay Clonogenic assay Analysis of apoptosis Western blot analysis Telomerase activity assay	Inhibited cell viability of both ovarian cancer cells time- and dose-dependently. Arrested the ovarian cancer cells in the G2/M phase. Down-regulated Bcl-2 (a gene involved in anti-apoptosis) protein levels in both cell types. Up-regulated the cleaved poly(ADP-ribose) polymerase (PARP) and phosphorylated H2AX. SFN + EGCG arrested cells in both the G2/M and S phase. SFN + EGCG increased apoptosis in SKOV3TR-ip2 cells, while reducing the expression of *hTERT.*	[132]
	Purchased	Prostate cancer TRAMP C1 cells DNA extraction Bisulfite genomic sequencing Methylation DNA immunoprecipitation (MeDIP) analysis RNA isolation and qPCR Western blot analysis ChIP assay	Decreased methylated CpG ratio in the promoter region of Nrf2 gene in TRAMP C1 cells. Decreased the binding of anti-methyl cytosine antibody to the promoter region of Nrf2 gene in TRAMP C1 cells. Induced the expression of Nrf2 and its downstream gene. Altered the expressions of epigenetics modifying enzymes.	[126]
	Purchased	Primary pancreatic ductal adenocarcinoma (PDA) cell line and non-malignant pancreatic ductal cells MTT assay Apoptosis measurement Detection of ALDH1 activity Western blot analysis Detection of MMP-2, MMP-9, and K-ras mRNA expression Detection of microRNA expression	Inhibited colony formation. Sulforaphane + EGCG inhibited viability, migration, expression of MMP-2 and -9, ALDH1 activity, colony, and spheroid formation and induced the apoptosis. Sulforaphane + EGCG induced the expression of miR-let7-a in cancer cells.	[133]
	Purchased	JB6 P+ cells Western blot analysis RNA isolation Quantitative real-time PCR DNA isolation Bisulfite genomic sequencing HDAC activity assay	Increased Nrf2 nuclear translocation and protein expression, up-regulating the mRNA and protein expression of Nrf2 target enzymes in JB6 P+ cells. Inhibited TPA-induced JB6 P+ cell transformation. Increased relative Nrf2 mRNA expression when cells are treated with TPA. Decreased the methylation status of the Nrf2 gene promoter. Down-regulated the epigenetic modifying enzymes in JB6 P+ cells.	[134]
	Purchased	Prostate cancer cells (LnCAP and PC3) ChIP assay Sample preparation for DNA methylation array DNA methylation array data analysis Validation of DNA methylation data Gene expression analyses	SFN + DIM decreased DNMT gene expression and caused distinct DNA methylation profile alterations depending on prostate cell line. SFN + DIM reversed cancer-associated DNA methylation alterations in LnCAP cells.	[16]
	Purchased	Cervical carcinoma cell line (HeLa) DNMT and HDAC activity assay In Silico molecular modeling studies of DNMT3B and HDAC1 Bisulfite Modification and MSP RT-PCR	Inhibited the DNMTs activity and down-regulated the expression of DNMT3B in HeLa cells. Inhibited the HDACs activity and reduced the expression of HDAC1 in HeLa cells. Interacted with DNMT3B and HDAC1. Reactivated or increased the expression of RARβ, CDH1, DAPK1, and GSTP1 genes in HeLa cells.	[135]
	Purchased	Breast cancer cells (MCF-7 and MDA-MB-231) Cell growth and apoptosis assays Methylation-sensitive restriction analysis Quantitative real-time PCR	Inhibited breast cancer cell growth and enhanced the clofarabine (ClF) inhibitory effect on cell viability. Induced the apoptosis in breast cancer cells and enhanced ClF Pro-apoptotic effects. Induced the hypomethylation of PTEN and RARbeta2 promoters with concomitant gene up-regulation. Increased epigenetic effects of ClF at non-invasive stages of breast cancer development. Induced p21 without altering DNMT1 expression.	[91]
	Purchased	Human lung cancer cells (A549 and H1299) HDAC activity assay Western blot analysis qRT-PCR Apoptosis assay Cell cycle analysis Tumor xenografts	Inhibited HDAC activity in lung cancer cells. Increased the acetylation of histones H3 and H4. Increased cell cycle and apoptotic-related gene expressions. Induced cell death and inhibited cell cycle arrest at S and G2/M in lung cancer cells. Suppressed the growth of A549 xenografts and HDAC activity in vivo.	[136]
	Purchased	Human breast cancer cells MCF-7, MDA-MB-231, and SK-BR-3 MTT assay Cell cycle analysis Apoptosis assay Genotoxicity and DNA damage response Western blot analysis Real-time PCR Global DNA methylation N^6^-methyladenosine (m^6^A) RNA methylation microRNA profiling	Promoted cell cycle arrest, elevation in the levels of p21 and p27, and cellular senescence (5–10 μM). Induced apoptosis (at 20 μM) Stimulated the energy stress as judged by decreased pools of ATP and AMPK activation and autophagy induction. Induced the global DNA hypomethylation. Decreased the levels of DNMT1 and DNMT3B. Diminished pools of m^6^A RNA methylation. Decreased the levels of miR-23b, miR-92b, miR-381, and miR-382 in breast cancer cells.	[137]
	Not reported	Breast cancer cells (MCF-7 and MDA-MB-231) Cell density assay MTT assay RNA isolation qRT-PCR Western blot analysis DNMTs and HDACs activity assay	SFN + WA promoted cell death. SFN + WA decreased the HDAC expression and promoted varying changes in DNMT expression. SFN + WA induced the changes in BAX and BCL-2.	[138]
	Purchased	Breast cancer cell line MCF-7 ChIP assay UPLC-Orbitrap-MS Immunofluorescence assay	Restored the alanine and lactic acid levels. Increased the levels of 4-OCH_3_E_2_. Influenced the expression of *COMT* through methylation mechanisms. Reversed the E_2_-induced methylation status. Epigenetic modulation of *COMT* expression subsequently influenced E_2_ metabolism.	[139]
	Purchased	Lung adenocarcinoma (A549), embryonic kidney (HEK293), and colorectal adenocarcinoma (HT29) cell lines Cell viability assay Bisulfite genome sequencing MeDIP analysis RNA extraction Quantitative real-time PCR Western blot analysis ChIP assay HDAC and DNMT activity assays	Decreased CpG methylation in the promoter region of miR-9-3. Increased H3K4me1 enrichment at the miR-9-3 promoter. Induced miR-9-3 expression. Diminished the expression and activity of epigenetic modifying enzymes. Decreased the protein expression of CDH1.	[140]
	Not reported	Human breast cancer cells Quantitative real-time PCR Western blot analysis HDACs activity assay Global histone H3 acetylation quantification Global DNA methylation analysis RNA sequencing analysis	SFN-based broccoli sprout diet induced: Decreased tumor incidence and inhibited early breast cancer development. Increased gene transcription in tumor suppressor genes such as p53 and p16^INK4a^. Decreased expressions of tumor-promoting genes such as *TERT* and *c-Myc*. Decreased gene expression and enzymatic activity of HDAC1, but did not affect DNMT1 gene expression. Decreased global DNA methylation level. Increased histone acetylation levels in two important histone acetylation markers (histone acetyl-H3K9 and acetyl-H3K14).	[141]
	Not reported	Human breast cancer MCF-7 and MDA-MB-231 cells Cell viability assay Methylation-sensitive restriction analysis Quantitative real-time PCR (MSRA)	SFN + clofarabine reactivated the DNA methylation- silenced CDKN2A tumor suppressor. SFN + clofarabine inhibited cancer cell growth at a non-invasive breast cancer stage.	[142]
	Not reported	Colon cancer (HCT116 and RKO) cells MTT assay Cellular density assay RT-PCR Cell cycle analysis Western blot assay HDAC activity assay	Decreased cell density. Inhibited the cell viability. Induced the apoptosis. Down-regulated the oncogenic miR-21, HDAC and *hTERT* mRNA and protein and enzymatic levels in colorectal cancer cells.	[143]
	Purchased	Breast cancer cells (MCF-7 and MDA-MB-231) MTT assay RNA isolation Quantitative real-time PCR Cell cycle analysis Western blot analysis HDAC activity assay Histone methyltransferase (HMT) activity assay	SFN + GEN decreased cell viability of breast cancer cell lines. SFN + GEN increased the rate of apoptosis and lowered the colony formation potential of cells. SFN + GEN inhibited cell cycle progression to the G2 phase in MDA-MB-231 and G1 phase in MCF-7 breast cancer cell lines. SFN + GEN inhibited HDAC and HMT. SFN + GEN down-regulated the levels of HDAC2 and HDAC3 both at the mRNA and protein levels. SFN + GEN down-regulated the *hTERT* levels.	[144]
	Purchased	Breast cancer cell lines (MCF-7 and MDA-MB-231) Flow cytometry cell cycle analysis DNA extraction Quantitative RT-PCR Western blot analysis Global methylation activity assay Histone acetyltransferase activity/Inhibition assay Histone methyltransferase activity/Inhibition assay ChIP assay	SFN + WA regulated cell cycle progression from the S to the G2 phase through the inhibition of cell cycle genes in breast cancer cells. SFN + WA promoted changes in epigenetic regulators in MCF-7 and MDA-MB-231 cells. SFN + WA promoted changes in p53 and p21 in breast cancer cells.	[138]
	Not reported	Nasopharyngeal carcinoma (NPC) cells, C666-1 Cancer stem cell (CSC) Cell viability analysis Flow cytometric analysis Real-time PCR Small interfering RNA (siRNA)-mediated silencing method Western blot analysis Nude mice tumorigenicity assay	Inhibited the formation of CSC-enriched NPC tumor spheres. Reduced the population of cells with CSC-associated properties. Restored the expression of Wnt inhibitory factor 1 (WIF1). Down-regulated DNMT1 activity. Inhibited the in vivo growth of C666-1 cells. Enhanced the anti-tumor effects of cisplatin.	[145]
	Not reported	Mouse melanoma B16F10 cells Cell cycle arrest analysis Apoptotic analysis RNA-Seq analysis Histone PTM mass spectrometry	SFN and DAC single and combination treatment resulted in growth inhibition. SFN and DAC single and combination treatment resulted in minimal apoptosis. SFN and DAC single and combination treatment resulted in no cell cycle arrest. SFN induced dysregulated gene transcription. SFN and DAC uniquely induced dysregulated gene transcription. No significant alterations detected in histone epigenetic modifications.	[146]
	Not reported	Neural crest cells (NCCs), JoMa1.3 cells Quantitative real-time PCR Western blot analysis ChIP-qRT-PCR analysis Snail1 siRNA transfection Analysis of apoptosis	Reduced ethanol-induced apoptosis. Diminished ethanol-induced changes in the expression of E-cadherin and vimentin. Restored the EMT (epithelial-mesenchymal transition) in ethanol-exposed NCCs. Diminished the ethanol-induced reduction of H3K4me3 at the promoter regions of the Snail1 gene. Restored the expression of Snail1. Down-regulated the Snail1 target gene E-cadherin.	[147]
	Not reported	Hepatocarcinoma cells, HepG2 and human primary gastric cells, GAS RNA-Seq analysis Cell cycle arrest analysis	Induced cytotoxic effects and reduced the cell viability in both cell lines at higher concentrations. Induced cell cycle arrest in G2/M. Increased the expression of cyclin-dependent cyclins and kinases (CDK). Induced DNA damage in HepG2. Inhibited HDACs activity. Down-regulated the chromatin profile controlling enzymes. Induced the apoptosis and triggered pro-apoptotic signals in both cell lines. Down-regulated the MAPK/ERK/JUN and PIK3/AKT signaling pathways. Altered the methylation pattern in both strains.	[148]
	Purchased	Caco-2 human colon adenocarcinoma cells DNA extraction and C-T conversion MSP RT-qPCR DNMT1 enzyme activity detection Western blot analysis	No effect on the DNMT1 mRNA expression levels. SFN and 5-Aza + TSA inhibited DNMT1 protein expression. Decreased Nrf2 promoter methylation. Inhibited Nrf2 protein expression.	[149]
	Purchased	Human hepatocarcinoma cell line, HepG2 Cell viability assays RNA extraction RNA-Seq analysis Apoptosis detection by flow cytometry Cell cycle analysis by flow cytometry	Reduced the viability of HepG2 cells. Induced DNA damage, mitotic spindle abnormalities, apoptosis, and proliferation inhibition in HepG2 cells. Up-regulated the DNA damage response and cell cycle checkpoint genes. Down-regulated the pathways frequently overexpressed in human cancer. Inhibited HDACs activity. Affected the activity of oncogenic TF through methylation of its binding sites motifs.	[150]

**Table 4:** Anticancer effects of quinones with epigenetic targets.

Bioactive Molecules	Origin	Experimental Methods	Key Results	References
d-antroquinonol	Not reported	Breast cancer cells (MCF-7, T-47D, and MDA-MB-231) DNMT1 enzyme activity assay Molecular modelling and docking Illumina methylation 450K array-based assay Real-time RT-PCR Western blot analysis Growth inhibition assay	Inhibited DNMT1 in a dose-dependent manner but not DNMT3B. Bind to the catalytic domain of DNMT1. Decreased the methylation level of multiple TSGs, including the FANCC and CACNA1A genes. Increased the FANCC, CACNA1A mRNA, and protein expression levels. Inhibited the growth of breast cancer cells.	[160]
	*Antrodia camphorate*	Non-small cell lung cancer cell lines DNMT enzyme activity assay Cell viability and migration ability From flow cytometry analysis	Inhibited the cell migration ability of CL1-5 cells. Induced high cytotoxicity toward different lung cancer cell lines. Up-regulated cyclin D2 gene expression in CL1-5 cells in time-dependent and dose-dependent manners. Caused cell cycle arrest at the G0/G1 phase.	[161].
	*Antrodia camphorata*	Breast cancer cell lines (MCF7, T47D, and MDA-MB-231) Molecular modeling and docking DNMT1 and DNMT3B methyltransferase activity assays MTT assay DNA methylation assay Real-time RT-PCR Western blot analysis	Inhibited the growth of MCF7, T47D, and MDA-MB-231 breast cancer cells. Inhibited the migratory ability of MDA-MB-231 breast cancer cells. Inhibited the DNMT1 activity. Bind to the catalytic domain of DNMT1. Decreased the methylation status and reactivated the expression of multiple TSGs in MDA-MB-231 breast cancer cells.	[162]
Emodin	Purchased	Human bladder urothelial cell carcinoma Cell viability (MTT assay) Western blot analysis Semi-quantitative-PCR, quantitative real-time PCR cDNA microarray analysis ChIP assay	Inhibited the cell growth of four bladder cancer cell lines in a dose- and time-dependent manner. Altered the epigenetic modifications. Suppressed pH3Ser10 and increased H3K27me3, contributing to gene silencing in bladder cancer cells. Repressed the oncogenic genes. Increased H3K27me3 and decreased pH3Ser10 modifications on the promoters of repressed genes, indicating that emodin reverses the cancer epigenetics towards normal epigenetic situations.	[163]
	Purchased	Human pancreatic cancer cell line PANC-1 Cell proliferation assay Dot-blot assay mRNA-sequence BSP assay Real-time PCR Western blot analysis	Inhibited the growth of pancreatic cancer PANC-1 cells in a dose- and time-dependent manner. Inhibited the genomic 5mC expression in the PANC-1 cells. Altered the gene expression profile in the PANC-1 cells. Decreased the methylation levels of P16, RASSF1A, and ppENK. Increased the unmethylated status.	[164]
	Not reported	Forty golden Syrian hamsters Tumor induction in the buccal pouches Western blot analysis	Inhibited tumor formation. Reduced the severity of precancerous pathological lesions. Corrected the abnormalities in the expression pattern of Akt, MAPK, ERK, and DNMT in the buccal mucosa.	[165]
	Purchased	Human pancreatic cancer cell line PANC-1 Cell proliferation assay Dot-blot assay BSP assay Fluorescent quantitative PCR (FQ-PCR) Western blot analysis	Inhibited the growth of pancreatic cancer Panc-1 cells in a dose- and time-dependent manner. Caused slight demethylation. Emodin + 5-Aza-CdR significantly suppressed the expression of genome 5mC in PANC-1 cells. Emodin + 5-Aza-CdR induced more significant demethylation. Emodin + 5-Aza-CdR increased the expression levels of P16, RASSF1A, and ppENK more significantly. Emodin + 5-Aza-CdR reduced the expression levels of DNMT1 and DNMT3a more significantly. Emodin + 5-Aza-CdR enhanced the demethylation effect of 5-Aza-CdR by reducing the expression of methyltransferases.	[166]
	Purchased	Lymphoma Raji cells Cell proliferation assay Flow cytometry Total RNA isolation and RT-qPCR analysis Luciferase reporter assay	Decreased the percentage of Raji cell viability. Induced apoptosis. Increased the activation of caspase 3, caspase 9, and poly (ADP-ribose) polymerase through the downregulation of ubiquitin-like proteins containing PHD and RING domains 1 (UHRF1). Increased the level of DNMT3a, which inhibited the activity of p73 promoter 2 and decreased the levels of NH2-terminally truncated dominant-negative p73.	[167]
	Not reported	Human breast cancer cell lines MDA-MB-453, MDA-MB-231, and MCF-7 Qpcr Western blot assay of hTERT, c-myc, and E2F1 proteins Methylation analysis by bisulfite modification	Induced the telomere shortening and telomerase inhibition. Induced a demethylation of CpG islands in *hTERT* gene promoter in MDA-MB-453 and MCF-7 cells. Decreased the transcription of *hTERT* gene in the three breast cancer cell lines via the up-regulation of E2F1 and down-regulation of c-myc expressions.	[168]
Laccaic acid (LA)	Purchased	Human colon carcinoma cell line HT29 Induced colon cancer rat model Cell viability assay Cell cycle arrest analysis Determination of DNMT1, HDAC1, TNF-α, IL-6 levels	LA + PEITC reduced the cell viability with apoptotic cell death (in vitro). LA + PEITC attenuated the number of aberrant crypt foci, fecal consistency score, IL-6, TNF-α, DNMT1, and HDAC1 levels (in vivo).	[129]
Physcion 8-O-β-glucopyranoside (PG)	Not reported	Human HepG2 cells Cell viability assay Cell cycle analysis Cell apoptosis assay Overexpression of DNMT1 and Sp1 RT-PCR assay Western blot analysis	Inhibited the growth and suppressed the invasion of HepG2 cells by down-regulating DNMT1 via ROS-dependent AMP-activated protein kinase (AMPK)- mediated modulation of transcription factor Sp1	[169]
	Not reported	Human breast cell line (MDA-MB-231) qRT-PCR Establishment of DNMT1- and Sp1-ovexpressing cell lines Knockdown of DNMT1 or Sp1 in MDA-MB-231 cells Western blot analysis In vivo lung metastasis model	Inhibited the MDA-MB-231 cell proliferation. Inhibited the EMT process in MDA-MB-231 cells. Suppressed the DNMT1 expression via AMPK/Sp1 signaling. Reduced the lung metastasis of MDA-MB-231 cells in animal models.	[170]
	Not reported	Testicular germ cell tumors (TGCTs) NCCIT and NTERA2 Cell cycle analysis Cell apoptosis assay RT-qPCR Western blot analysis Tumor induction in a xenograft mouse model	Inhibited NTERA2 and NCCIT cell proliferation, blocked the cell cycle, and induced cell apoptosis. Suppressed LDH release, glucose consumption, lactate production, and ATP generation in NTERA2 and NCCIT cells. Increased the miR-199a expression in TGCTs Inhibited the tumor growth in vivo.	[171]
Shikonin	Purchased	MCF-7 and HeLa cells Luciferase reporter assay DNA fragmentation assay Cell proliferation assay	Regulated p73, p16^INK4A^, ICBP90, and DNMT1 expression. Increased the p16^INK4A^ promoter activity through the down-regulation of ICBP90. INK4A Induced the apoptosis in MCF-7 and HeLa cells. Induced the apoptosis via a caspase-dependent mechanism.	[172]
	Not reported	Human papillary thyroid cancer (PTC) cell line, TPC-1 Cytotoxicity assay DNMT1 gene knockdown and overexpression Transwell cell migration and invasion assay DNA extraction and MSP assay Western blot analysis	Decreased the cell survival rate of TPC-1 cells in a dose-dependent manner. Inhibited the TPC-1 cell migration and invasion in a dose-dependent manner. Suppressed the methylation of PTEN, which reduced the expression of DNMT1 in a dose-dependent manner, and increased the expression of PTEN. Decreased the levels of protein expression of PTEN in TPC-1 cells.	[173]
Naphthazarin (Naph)	Purchased	Human breast cancer cell line, MCF-7 Cell proliferation assay and cell morphology RNA isolation and quantitative real-time PCR Western blot analysis ChIP assay Cell cycle analysis Apoptosis analysis	Reduced the MCF-7 cell viability in a dose-dependent manner Naph + IR (ionizing radiation) increased the p53-dependent p21 (CIP/WAF1) promoter activity. Naph + IR activated the p21 promoter via the inhibition of binding of multi-domain proteins, DNMT1, UHRF1, and HDAC1. Naph + IR induced cell cycle arrest and apoptosis in MCF-7 cells.	[174]
Nanaomycin A	Purchased	A549, HL60, and HCT116 cells DNA methylation analysis Methylation analysis of the RASSF1A promoter region RNA isolation and quantitative real-time PCR Western blot analysis Biochemical DNMT assay Molecular docking	Reduced the global methylation levels in all three cell lines. Reactivated the transcription of the *RASSF1A* tumor suppressor gene. Revealed a selectivity toward DNMT3B.	[174]
Thymoquinone	Purchased	Human leukemic T-cell line Jurkat (clone E6-1) Cell proliferation, viability, and apoptosis assays Cell cycle phase distribution analysis and quantitation of hypodiploid sub-G0/G1 cell population Assessment of DNA fragmentation pattern Western blot analysis	Inhibited cell growth and induced cell cycle arrest of Jurkat cells. Induced the apoptosis in Jurkat cells. Induced the generation of ROS and the breakdown of ΔΨm in Jurkat cells. Up-regulated the p73 and down-regulated the UHRF1 in Jurkat cells. Induced the apoptosis, cell cycle arrest, and deregulation of p73 and UHRF1 expressions in Jurkat cells, which induced apoptosis via a caspase-dependent mechanism.	[175]
	Purchased	Human acute lymphoblastic leukemia (ALL) p53-mutated cells, Jurkat cells (clone E6-1) Cell apoptosis and proliferation assays Cell cycle analysis Western blot analysis	Induced an initial down-regulation of PDE1A in the acute lymphoblastic leukemia Jurkat cell line with a subsequent down-regulation of UHRF1 via a p73-dependent mechanism.	[176]
	Purchased	Human cancer cell lines MDA-MB-435, HeLa and BT549, and mouse breast cancer cell line 4T1 Cell growth, migration, and invasion assay RNA extraction, RT-PCR, and qPCR analysis Protein extraction and western blot analysis Generation of breast tumor model of mouse Gene methylation assay	Inhibited cancer cell growth, migration, and invasion in a dose-dependent manner. Decreased the transcriptional activity of the TWIST1 promoter and the mRNA expression of TWIST1, an EMT-promoting transcription factor. Decreased the expression of TWIST1-upregulated genes such as N-Cadherin and increased the expression of TWIST1-repressed genes such as E-Cadherin. Inhibited the growth and metastasis of cancer cell-derived xenograft tumors in mice but partially attenuated the migration and invasion in TWIST1-overexpressed cell lines. Enhanced the promoter DNA methylation of the TWIST1 gene in BT 549 cells.	[177]
	Purchased	Cell lines, Kasumi-1, MV4-11, THP-1, and ML- 1 Leukemia- bearing mice Human DNMT1 homology modeling Docking simulation In vitro enzymatic activity assays Quantification of DNA methylation Colony formation and flow cytometry assays ChIP assay Western blot analysis RNA isolation and qPCR	Interacted with the catalytic pocket of DNMT1 and competed with co-factor SAM/SAH for DNMT1 inhibition. Decreased the DNMT1 methylation activity in a dose-dependent manner with an apparent IC_50_ of 30 nM. Down-regulated the DNMT1, mechanistically, through dissociation of Sp1/NFkB complex from DNMT1 promoter. Reduced DNA methylation. Decreased the colony formation and increased the cell apoptosis via the activation of caspases. Induced leukemia regression.	[178]
	Purchased	Human T lymphocyte cell line Jurkat, HL60, and HeLa cell lines Western blot analysis Apoptosis assays Real-time RT-PCR analysis	Induced the degradation of UHRF1 (Ubiquitin-like containing PHD and Ring Finger 1), correlated with a sharp decrease in HAUSP (herpes virus-associated ubiquitin-specific protease) and an increase in cleaved caspase-3 and p73. Rapid ubiquitination of UHRF1, concomitantly.	[179]
	Purchased	T-cell ALL JK cell line and MDA-MB-468 cell line, a human epithelial breast cancer cell line Cell proliferation assay RNA-seq and differentially expressed gene analysis Apoptosis assay Real-time RT-PCR analysis	Down-regulated many key epigenetic players, including ubiquitin-like containing plant homeodomain (PHD) and really interesting new gene (RING) finger domains 1 (UHRF1), DNMT1,3A,3B, G9A, HDAC1,4,9, KDM1B, and KMT2A,B,C,D,E in Jurkat cells. Up-regulated several TSGs, such as DLC1, PPARG, ST7, FOXO6, TET2, CYP1B1, SALL4, and DDIT3. Up-regulated several downstream pro-apoptotic genes, such as RASL11B, RASD1, GNG3, BAD, and BIK.	[180]

## Data Availability

Not applicable.
